# Association of Qualitative Characters With Agronomic Traits, and Their Breeding Importance in Lentil (*Lens culinaris* Medikus)

**DOI:** 10.1002/pei3.70162

**Published:** 2026-05-13

**Authors:** Nigussie Kefelegn, Solomon Benor, Gizachew Haile, Asnake Fikre, Ming Pei You, Martin J. Barbetti

**Affiliations:** ^1^ Department of Biotechnology Addis Ababa Science and Technology University Addis Ababa Ethiopia; ^2^ Crop Research Directorate, Debre Berhan Agricultural Research Centre Debre Berhan Ethiopia; ^3^ Department of Plant Biology and Biodiversity Management, College of Natural and Computational Sciences Addis Ababa University Addis Ababa Ethiopia; ^4^ Department of Industrial Engineering, Faculty of Engineering and the Built, Environment Tshwane University of Technology Pretoria South Africa; ^5^ Debre Zeit Agricultural Research Centre Debre Zeit Ethiopia; ^6^ School of Agriculture and Environment and the UWA Institute of Agriculture, University of Western Australia Crawley Western Australia Australia

**Keywords:** agronomic traits, association, characters, lentil, qualitative traits

## Abstract

There is a paucity of available information on lentil ( *Lens culinaris* Medikus) qualitative traits and their associations with agronomic traits, despite such information being essential for the selection of best genotypes. Hence, this study was undertaken to evaluate relationship among qualitative and agronomic traits, and to explore the significance of these relationships for lentil breeding programs. One hundred ninety‐two lentil germplasm were evaluated in 2025 at Dogolo and Enewari in the Amhara Region of Ethiopia. Pot experiment was also undertaken at Debre Berhan Agricultural Research Centre for two consecutive years (2024 and 2025). Alpha lattice experimental design was used and data on 24 agro‐morphological traits were collected. Chi‐square tests, Cramér's *V*, Spearman's rank correlation, point‐biserial correlation, and principal component analysis were employed to assess relationships and effect sizes among qualitative and agronomic traits. Additionally, Welch's *t*‐test and the Welch's ANOVA were used to compare the mean values of agronomic traits across different groups. Large association was observed among seed and flower characters (Cramer (*V*) = 0.61 to 0.76), and also seed with leaf related characters (*V* = 0.30 to 0.35) across both experiments. On the other hand, leaf pubescence showed a strong association with days to maturity (*ρ* = 0.41–0.53), as well as with seed (*ρ* = −0.27 to −0.35) and pod (*ρ* = −0.23 to −0.35) development across both experiments. Mean comparison also showed that germplasm with medium leaf pubescence tend to have longer time to mature while producing fewer seeds and pods per plant compared to those with absent or slightly dense pubescence. Seed pigmentation pattern, cotyledon and flower color also showed a larger effect size on the phenology, seed size and yield traits where highly pigmented type tended to mature earlier and produce smaller seeds but showed improved yield performance. Overall, this study demonstrates that strong associations exist between several qualitative and agronomic traits, which provide valuable insights for breeders in the selection of desirable traits for adaptation and variety development, and also offer a useful basis for further studies on linkage analysis, association mapping, underlying molecular mechanisms controlling those traits in diverse lentil germplasm.

## 1 Introduction

Lentil ( *Lens culinaris* Medikus) is an annual herbaceous cool‐season legume with a wide range of morphological variation in both vegetative and reproductive traits. The considerable variation among the traits used in lentil breeding and selection programs has been widely reported (Ahamed et al. 2014; Cristóbal et al. 2014; Saxena 2009). A previous study by Kefelegn et al. (2024) highlighted enormous variation in terms of plant vigor, stem pigmentation, growth habit, leaf color, leaf pubescence, leaf size, seed and flower characteristics. These authors also showed a variation in agronomic traits among the lentil germplasm grown worldwide and conserved in different gene banks.

Improvements in lentil breeding programs are dependent on an effective selection regime based across different desirable traits and their associations. Seed yield is considered the ‘ultimate’ trait and is an outcome of the interaction of these desirable traits (Dalbeer et al. 2015). The genetic architecture of seed yield, not only in lentil but also in other crops, is based on the overall combined effect produced by various other traits. Identification of these traits and information about their associations, not only with yield but also with each other, is critical for selecting best genotypes towards developing high yielding varieties. Working with Bottle Gourd landraces, Mashilo et al. (2016) reported that understanding the nature of associations among and between qualitative characters, and with agronomic traits is essential for direct or indirect selection. Consequently, it leads towards improving the efficiency of selection gains in plant breeding programs. Knowledge on trait characteristics and their balanced and overall effect on seed yield or economic traits, as well as environmental adaptations, are prerequisites for targeted and more efficient utilization of germplasm by plant breeders (Bekele et al. 2023).

There is very limited research on lentil determining the association between qualitative and quantitative traits as well as within qualitative characters. However, Mashilo et al. (2016) showed that qualitative traits could be associated among each other and also with yield and yield‐related traits. Apart from direct and indirect interaction effect of qualitative and quantitative traits on lentil yield and environmental adaptation, they have a paramount role in quality, nutritional, and consumers' preference (Hussain et al. 2022; Matiur et al. 2009; Roy et al. 2012; Wilson and Hudson 1980). On the other hand, qualitative characters like stem, flower, cotyledon, and testa colors, and the pattern of testa and tendrils in lentil could be used as markers for testing the degree of hybridity and for maintaining genetic purity (Roy et al. 2012). Testa color and testa mottling are found to be the most stable and uniform traits for certification of genetic purity of lentil genotypes at the seed level as their expression is hardly influenced by the environment (Choudhary et al. 2017). With this background, the objective of this study was to evaluate the association among qualitative traits, and between qualitative and agronomic traits, as well as to explore the significance of these relationships for lentil breeding programs.

## 2 Materials and Methods

### 2.1 Research Site

**FIGURE 1:**
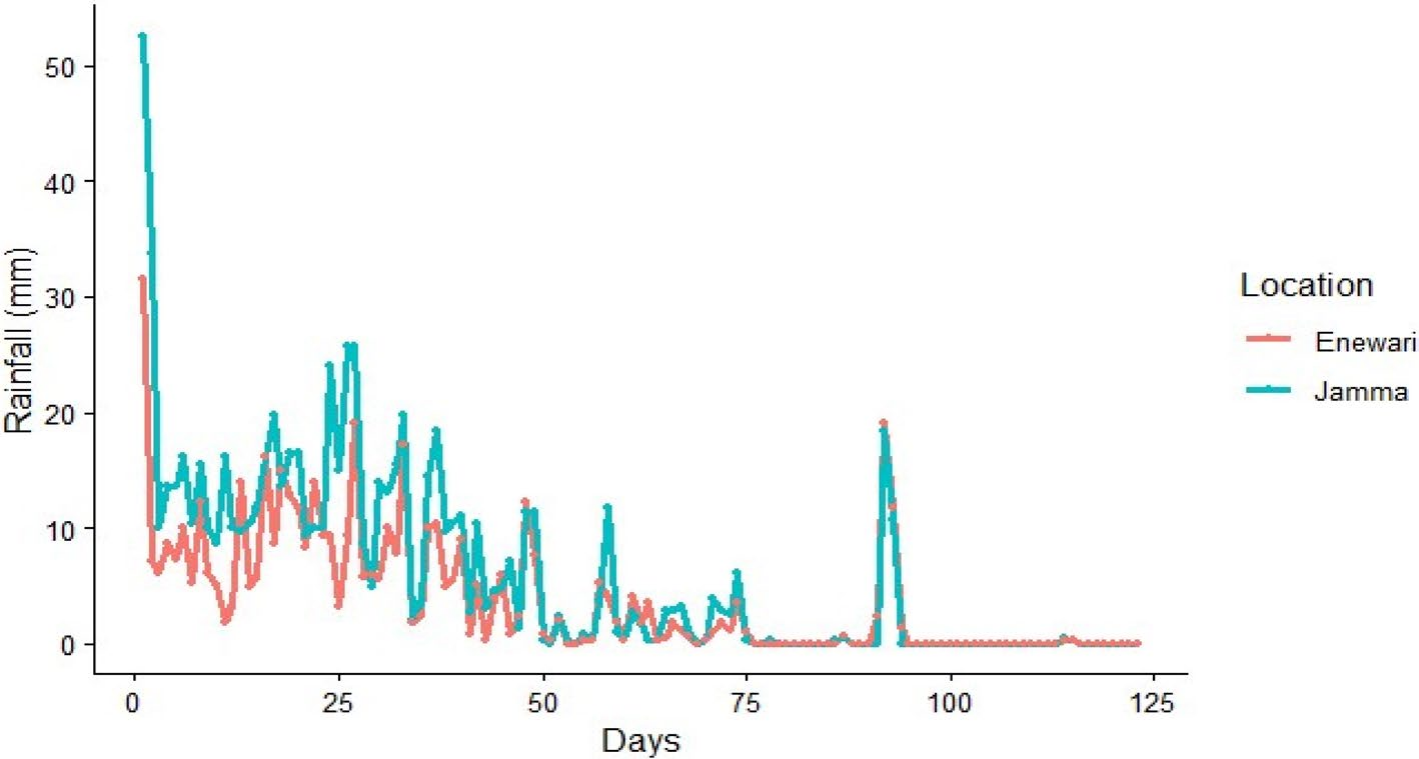
Rainfall distribution of study area during the growing period (*Source:* Amhara Region, Ethiopia Weather Trends | MSN Weather).

Field experiment was carried out at Jamma (Degolo, South Wollo, and Ethiopia) and at Moretina Jiru (Enewari, North Shewa, Ethiopia) in 2024. Degolo is 269 km north of Addis Ababa with an altitude of 2630 m.a.s.l. (GPS 10° 27′ North; 39° 15′ East). Enewari is located 195 km northeast of Addis Ababa with an altitude of 2665 m.a.s.l. (GPS 9° 52′ 10.7″ North; 39° 10′ 46.5″ East). Both are characterized by heavy vertisol soils and are major lentil producing areas. The daily average maximum and minimum temperatures of Dogolo ranges from 20.7°C to 9.8°C and at Enewari its range is between 18.9°C and 9.3°C during the growing season. Both locations always face terminal moisture deficit, especially on average after 76 days post sowing. The daily rainfall distribution during the growing period is depicted in Figure 1.

### 2.2 Planting Material

One hundred ninety‐two lentil germplasm advanced from the previous study by Kefelegn et al. (2024) were evaluated in the 2024 cropping season under rain‐fed conditions. The lentil germplasm consisted of varieties, landraces, and exotics from the Ethiopian Biodiversity Institute, the Australian Centre for International Agricultural Research (ACIAR), the International Center for Agricultural Research in the Dry Areas (ICARDA), and the Debre Zeit Agricultural Research Center (DZARC). List of planting material (germplasm) presented (Table S1).

### 2.3 Research Design

#### 2.3.1 Field

The field trial was laid out in an alpha lattice design with two replications so as to improve experimental precision by controlling field heterogeneity through incomplete blocking, and also since our experimental materials were a bit large. Each replication was arranged in 48 blocks, with 4 entries in each block. Each replication consisted of 24 beds of 0.8 m width, each bed having 2 blocks. Blocking was set parallel to the water flow in the experimental field so as to drain the excess water as well as to maintain the bed structure. Each lentil germplasm was planted with 2 replications randomly assigned to a separate single row with 0.4 m spacing and 4 m length on each bed (plot area = 1.6 m^2^). Between each bed there was a 0.4 m wide furrow for draining excess water which is a common practice in vertisol areas of Ethiopia. Spacing between blocks and replications was 1 m. One hundred seeds of each lentil germplasm were planted in a row and 8 g of NPS fertilizer based on EthioSIS (2014) recommendation (121 kg ha^−1^) was applied to each row. Sowing was done on 1st August 2024.

#### 2.3.2 Pots

Pot experiment was also carried out for the same set of materials at Debre Berhan Agricultural Research Center for two consecutive years of 2024 and 2025. Sowing was done in January for both years. Each pot contained a single germplasm, replicated twice and assigned randomly to each pot, and the pots were arranged in an alpha lattice design with two replications comprising 24 blocks, each block containing eight entries. Fifteen seeds were planted in each pot (30 cm diameter filled with vertisol) and 1 g of NPS fertilizer based on the recommended amount of (121 kg ha^−1^) was applied to each pot. All agronomic management for these experiments was adopted from the Ethiopian lentil research program. This pot experiment was conducted to validate the consistency and reliability of the findings in the field experiments.

### 2.4 Data Collected

For both experiments, all qualitative and quantitative data, as indicated in Table 1, were collected for all lentil germplasm. The lentil descriptor described in earlier studies was used for collecting these data (IBPGR and ICARDA 1985; Mohammed et al. 2019; Tripathi et al. 2021). However, qualitative data were collected for each germplasm both at pre‐harvest and post‐harvest stages according to the standard descriptors, as these traits are generally stable and less influenced by environmental factors (Serpico 2020).

**TABLE 1:** Descriptor to be used for morphological characterization of lentil germplasm.

Category of variable	Descriptors	Descriptor state	Code
Qualitative characters	Early plant vigor	Poor (1), Good (2), Very Good (3)	EPV
	Seedling stem pigmentation	Absent (0), Present (1)	SSP
	Growth habit	Erect (1), Semi‐erect (3), Horizontal (5)	GH
	Leaf color	Green (1), Light green (2), Pigmented (3)	LC
	Leaf pubescence	Absent (0), Slight (3), Medium (5), Dense (7)	LP
	Leaflet size	Small (3), Medium (5), Large (7)	LS
	Flower ground color	White (1), Yellow (2), Red (3), Purple (4)	FGC
	Lodging score	Absent (0), Present (1)	LoD
	Pod pigmentation	Absent (0), Present (1)	PP
	Seed coat color	Yellow (1), Green (2), Brown (3), Pink (4), Gray (5), Black (6)	SCC
	Pattern of seed testa	Absent (0), Dotted (1), Spotted (2), Marbled (3), Complex (4)	PST
	Cotyledon color	Yellow (1), Orange (2), Green (3)	CC
Quantitative (agronomic traits)	Days to 50% flowering	Plot basis	DF
	Secondary branches/plant	Average of five plants	SBPP
	Plant height (cm)	Average of five plants	PH
	Pods per plant	Average of five plants	PPP
	Seeds per plant	Average of 5 plants	SPP
	Days to 80% maturity	Plot basis	DM
	100‐seed weight (g)	Average of randomly sampled seeds	SW
	Biomass yield (g)	Plot/plant basis	BM
	Seed yield (g)	Plot/plant basis	YLD
	Harvest Index	Plot or plant basis	HI
	Seed diameter (mm)	Average of five seeds	SD
	Seed thickness (mm)	Average of five seeds	ST

*Note:* A single row for a single germplasm and calculated to be a plot size of 1.6^m^, BM = biomass data collected plot basis for field experiment and plant basis for pot experiment, YLD = yield data collected plot basis for field experiment and plant basis for pot experiment, HI = calculated from the ratio of yield to biomass multiplied by hundred.

### 2.5 Data Analysis

All statistical analysis in this study was carried out using R program (R development Core Team 2025). The combined dataset was analyzed using a mixed model (in *lme4 package*), treating germplasm as fixed and environment and its interaction with germplasm as random effects following the analysis of variance assumptions fulfilled on Bartlett's test and residual diagnosis (Figures S1–S10). Combined analysis was carried out separately for the two sets of experiments only on their quantitative or agronomic data; this is because the setup of the two experiments was quite different. All further analyses were conducted using the combined mean across locations for field experiments and across years for pot experiments.

#### 2.5.1 Statistical Analysis for Qualitative Traits

Descriptive statistics, Cramér's *V*, and non‐parametric tests, including the Chi‐square test of independence and Spearman rank correlation, were employed to analyze correlations among qualitative traits, as these methods do not require the assumption of normality.

#### 2.5.2 Chi Square Test

Chi square test is a non‐parametric test as described by Pearson (1900) and also used by Egbuchulem (2024) that was used under r package ‘rcompanion’ to determine whether the two categorical variables are independent (no association) or dependent (there is association). The equation of chi square test is described as follows:

$$\chi^{2}=\sum_{i=1}^{r}\sum_{j=1}^{c}\frac{{\left(O_{\mathrm{ij}}-E_{\mathrm{ij}}\right)}^{2}}{E_{\mathrm{ij}}}$$

where *χ* ^2^ = Chi‐square statistic; *O*_*ij*_ = Observed frequency in the *i*th row and *j*th column; *E*_*ij*_ = Expected frequency in that cell; *r* = number of rows; *c* = number of columns.

#### 2.5.3 Cramer—*V* Correlation Analysis

Cramer‐*V* function under rcompanion package was used to carry out correlation analysis between different categorical variables. Cramer's *V* is a statistical measure used to determine the strength of association between two categorical variables after performing a Chi‐square test of independence. It is based on the Chi‐Square test as described in Mchugh (2013).

$$V=\sqrt{\frac{{Χ}^{2}}{n\left(k-1\right)}}$$

*Χ* ^2^ = chi square value; *n* = Total sample size; *k* is the smaller value between the number of rows (*r*) and the number of columns (*c*) in the contingency table.

#### 2.5.4 Correlation Analysis Between Different Categorical Variables and Agronomic Traits

Spearman correlation coefficient was computed to determine the association between ordinal categorical variables and different agronomic traits strictly following the information provided in Khamis (2008). This is because Spearman's rank correlation does not require the strict assumptions that Pearson needs, and also used in previous study (Mashilo et al. 2016). The equation of spearman correlation (Spearman 1904) and also used by Sheskin (2000) as follow:

$$\rho =1-\frac{6\sum d^{2}}{n\left(n^{2}-1\right)}$$

where rho (*ρ*) spearman correlation; *d* difference between pair of observation; *n* number of paired observations.

The point‐biserial correlation (*r*_pb_) was used to compute the association between binary variables and agronomic traits as described by the previous studies (Sheskin 2000; Diana 2005).

$$\mathrm{rpb}=\frac{{\bar{Y}}_{1-}{\bar{Y}}_{0}}{{\bar{s}}_{y}}\sqrt{\frac{N_{1}N_{0}}{N\left(N-1\right)}}$$

where *Y*_0_ and *Y*_1_ are means of the metric observations for coded 0 and 1 respectively; *N*_0_ and *N*_1_ are number of observations coded 0 and 1 respectively; *N* is total number of observations = *N*_0_ + *N*_1_; and *s*_*y*_ is the standard deviation of all the metric observations. Finally, the effect size was estimated using Cohen's *d* to quantify the effect of binary variables on agronomic traits. The analysis was performed in R using the ltm package. The equation for the effect size is presented as follows:

$$d=\sqrt{\frac{N\left(N-2\right){r^{2}}_{\mathrm{pb}}}{N1N0\left(1-{r^{2}}_{\mathrm{pb}}\right)}}$$

In addition, Cohen's *f* was calculated to assess the magnitude of the association between polychotomous variables with more than two categories and the group means of agronomic traits, based on Cohen (1988), and it was computed using the effect size package in R. The model equation is as follows:

$$f=\frac{\sigma_{m}}{\sigma }$$

where *δ*_*m*_ is the standard deviation of the population means and *δ* is the within‐population standard deviation.

The benchmark of effect size of each categorical variable was determined based on Cohen (1988). Note that Cohen's *d* (for two groups) and Cohen's *f* (for multiple groups) were used to estimate standardized effect sizes in any data, so that not strictly required homogeneity of variance and normal distribution across groups as described in the previous study (Cohen 1988; Grissom and Kim 2005; Lakens 2013). However, our data showed approximately normal distribution within each group following the Shapiro–Wilk test.

For comparing means across different groups of categorical variables, Welch's *t*‐test (for two groups) and Welch's ANOVA (for three or more groups) were employed, as these tests are robust to unequal variances (Howel 2010; Mcgee 2025). The distribution of all traits was approximately normal, and observations were considered independent. To control for inflated Type I error due to multiple pairwise comparisons, *p*‐values from post hoc tests were adjusted using the Bonferroni correction.

#### 2.5.5 Principal Component Analysis

Principal component analysis was done under ‘FactoMineR package’ and used to illustrate the most important traits and their relationship in a two‐dimensional plane.

## 3 Results

### 3.1 Traits Heterogeneity

**FIGURE 2:**
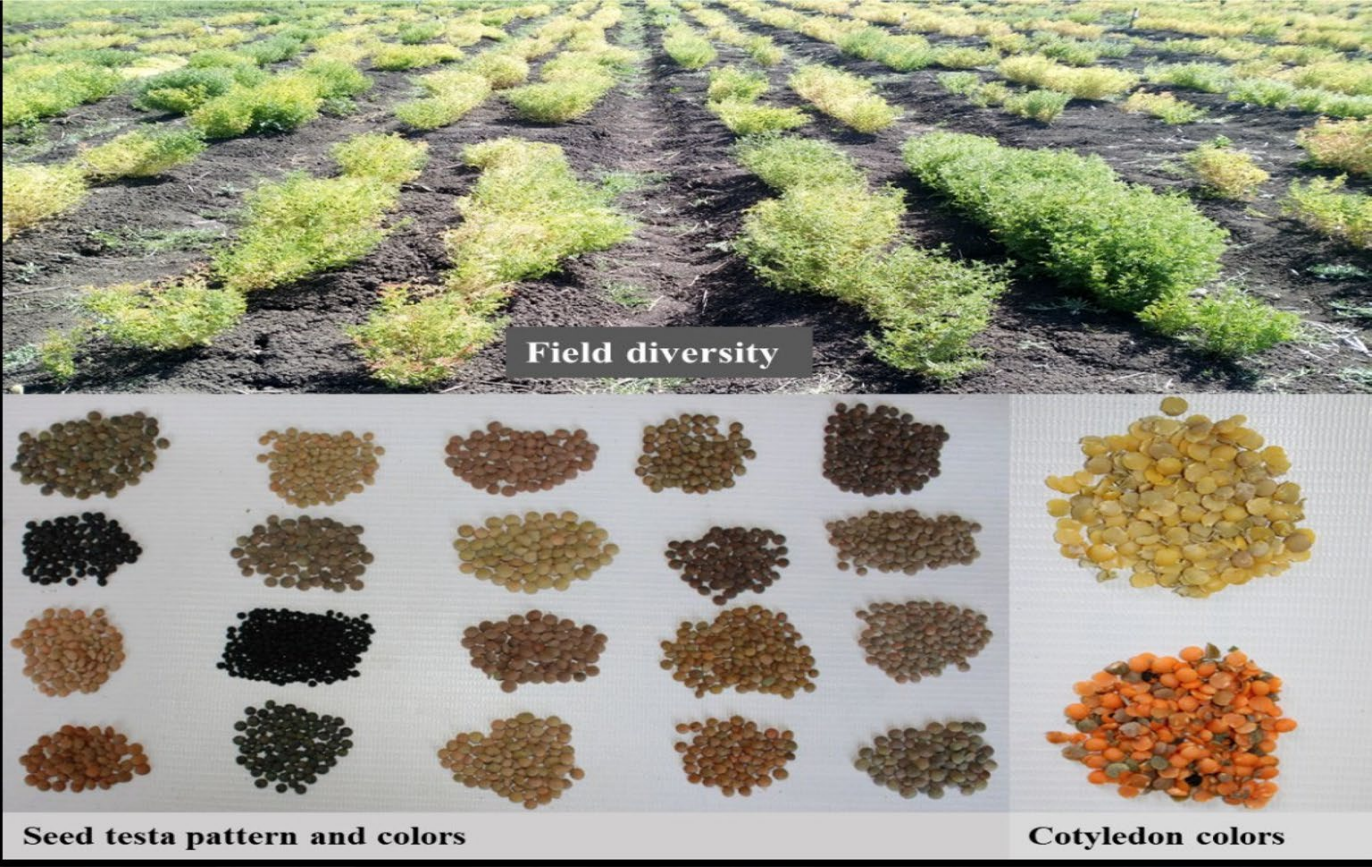
Examples of agro‐morphological diversity of evaluated lentil germplasm. Photo by Nigussie K. and Simegnew A., 2024, from field and laboratory.

The frequency distribution and the outcomes of the Chi‐square of goodness of fit test for qualitative characters for evaluated lentil germplasm is depicted in Table 2. The percentage distribution of the various qualitative characteristics recorded in this study showed statistical significance (*p* < 0.05) and hence the distribution of individuals significantly varied for all these characters across respective categories. The agro‐morphological diversity (field diversity, seed characters and cotyledon color) highlighted the obvious diversity of the lentil germplasm evaluated (Figure 2).

**TABLE 2:** Frequency distributions for each qualitative trait.

Qualitative character	Category	Frequency	Proportion	Percentage	*p*
Early plant vigor (EPV)	Good	88	0.46	45.83	
	Poor	51	0.27	26.56	0.001153
	Very good	53	0.28	27.60	
Seedling stem pigmentation (SSP)	Absent	82	0.43	42.71	0.04331
	Present	110	0.57	57.29	
Growth habit (GH)	Erect	112	0.58	58.33	0.02092
	Semi‐erect	80	0.42	41.67	
Leaf color (LC)	Green	122	0.64	63.54	0.0001749
	Light green	70	0.36	36.46	
Leaf Pubescence (LP)	Absent	48	0.25	25.00	
	Medium	55	0.29	28.65	1.24E‐7
	Slight	89	0.46	46.35	
Leaf size (LS)	Medium	113	0.59	58.85	2.05E‐9
	Small	79	0.41	41.15	
Flower ground color (FGC)	Purple	128	0.67	66.67	3.86E‐06
	White	64	0.33	33.33	
Lodging score (LoD)	Absent	87	0.45	45.31	0.1939
	Present	105	0.55	54.69	
Pod pigmentation (PP)	Absent	127	0.66	66.15	7.66E‐06
	Present	65	0.34	33.85	
Seed coat color (SCC)	Brown	102	0.53	53.13	2.25E‐16
	Gray	28	0.15	14.58	
	Green	62	0.32	32.29	
Pattern of seed testa (PST)	Absent	63	0.33	32.81	
	Complex	108	0.56	56.25	2.21E‐16
	Spotted	21	0.11	10.94	
Cotyledon color (CC)	Orange	160	0.83	83.33	2.20E‐16
	Yellow	32	0.17	16.67	

### 3.2 Association Among Categorical Traits

**FIGURE 3:**
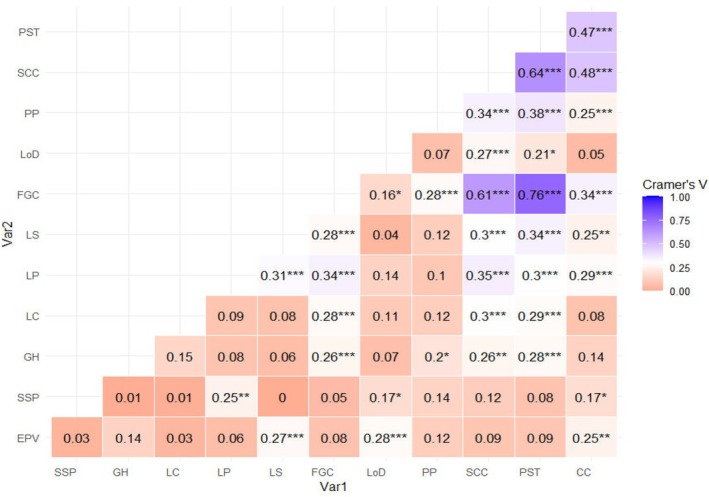
Cramer's *V* correlation heat‐map. 0.01–0.09 = Very Weak, 0.10–0.29 = Weak, 0.30–0.49 = Moderate, 0.50–0.69 = Very Strong Association following (Cohen 1988); CC, cotyledon color; EPV, early plant vigor; FGC, flower ground color; GH, growth habit; LC, leaf color; LP, leaf pubescence; LS, leaf size; PP, pod pigmentation; PST, Pattern of seed testa; SCC, seed coat color; SSP, seedling stem pigmentation. *Significant at the 0.05 probability level. **Significant at the 0.01 probability level.

The heat‐map (Figure 3) showed the pairwise associations among categorical traits based on Cramer's *V* statistics, which quantify the strength of association between categorical variables derived from the Chi‐square test of independence. The contingency table was presented in Table S2. Cramer's *V* values range from 0 (no association) to 1 (perfect association), with higher values indicating stronger associations between traits. Thus, the Chi‐square test indicates whether an association is statistically significant, whereas Cramer's *V* indicates how strong that association is. Although some trait pairs showed similar Cramer's *V* values, differences in significance levels were observed. This occurs because statistical significance in the Chi‐square test depends not only on the strength of association but also on factors such as sample size and the distribution of observations across categories. Consequently, associations with comparable effect sizes may yield different *p*‐values.

The results presented in the Cramer's *V* matrix indicate that most trait pairs exhibit weak to moderate associations, with values generally below 0.30. This suggests that although some statistically significant relationships may exist (as indicated by the Chi‐square test), the practical strength of association among most categorical traits is relatively limited. Consequently, many of the traits appear to represent distinct categorical characteristics rather than highly coinciding attributes. However, several trait combinations demonstrated relatively strong associations based on their Cramer's *V* values. In particular, FGC and PST (*V* = 0.76, *p* < 0.001) showed a strong association, followed by SCC and PST (*V* = 0.64, *p* < 0.001) and FGC and SCC (*V* = 0.61, *p* < 0.001). These values indicate that, in addition to being statistically significant, their relationships were also substantively strong. Furthermore, SCC and CC (*V* = 0.48, *p* < 0.001) and PST and CC (*V* = 0.47, *p* < 0.001) exhibited moderate associations. Both PST and SCC showed moderate (*V* = 0.30–0.35, *p* < 0.001) association with leaf related characters. FGC also moderately (*V* = 0.34, *p* < 0.001) associated with LP and CC. Moderate to strong association suggests a shared underlying categorical pattern among these categorical variables. It also indicates that these traits tend to occur together possibly because they are genetically linked as they are controlled by related genes or influenced by similar developmental pathways. This suggests the possibility of indirect selection, where improvement in one trait may lead to correlated improvement in another trait.

In contrast, most trait pairs showed weak associations (*V* < 0.30). Even if the Chi‐square test identifies statistical significance in some cases, such low Cramer's *V* values indicated that the strength of the association is negligible in practical terms. Thus, the combined interpretation of the Chi‐square significance test and Cramer's *V* effect size provides a comprehensive understanding of the relationships among the categorical traits. While the Chi‐square test identifies existence of associations, Cramer's *V* clarifies their magnitude. In the current study, it revealed that only a few trait pairs exhibit strong categorical associations, while most traits remained relatively independent.

### 3.3 Spearman Rank Correlation Analysis Between Ordinal Variables and Agronomic Traits

**FIGURE 4:**
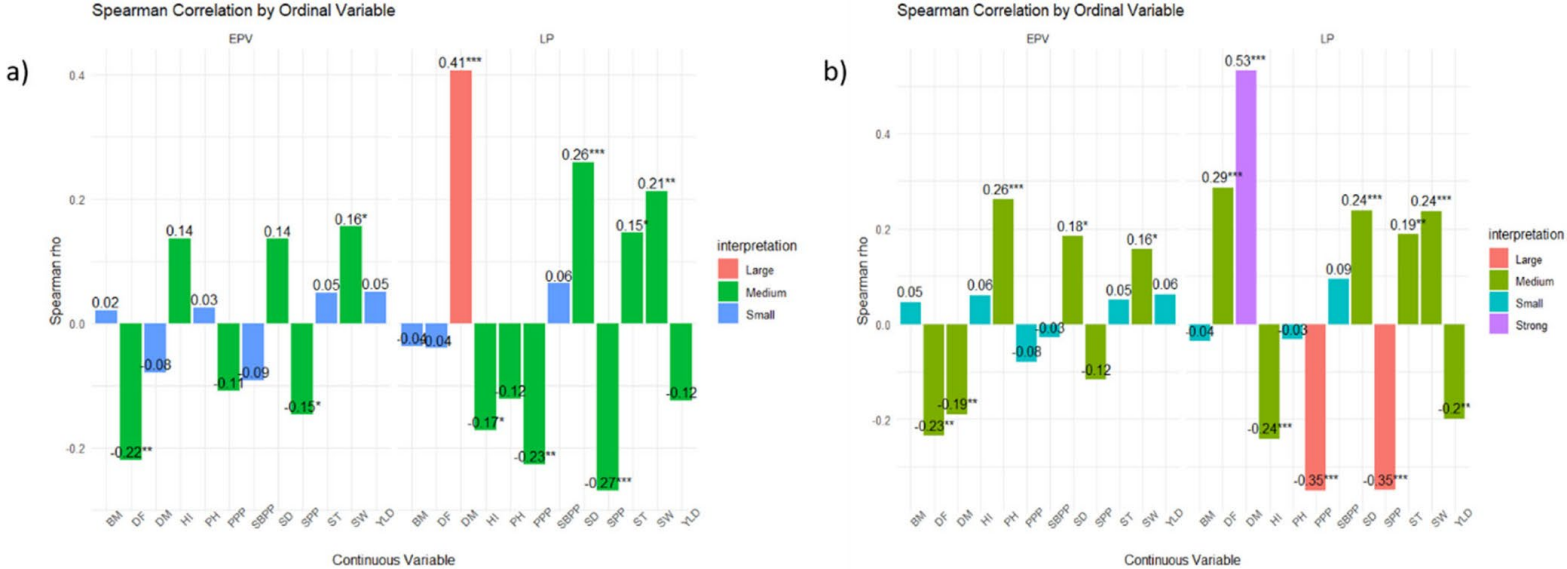
Spearman rank correlation coefficients illustrate the correlations between measured continuous variables (*x*‐axis) and ordinal variables (EPV, LP). (a) Represent field experiment and (b) represent pot experiment. The bars represent the magnitude of correlation explained by group membership for each agronomic trait. Correlation strength is outlined as small (|*ρ*| ≤ | 0.10), medium (0.10 ≤ |*ρ*| ≤ 0.30), large (0.30 ≤ |*ρ*| < 0.50), and strong (|*ρ*| ≥ 0.50) following (Cohen 1988). BM, biomass yield in grams; DF, days to flowering; DM, days to maturity; HI, Harvest index; PPP, number of pods per plant; PST, Pattern of seed testa; SBPP, number of secondary branches per plant; SCC, Seed coat color; SD, seed diameter in mm; SPP, number of seeds per plant; ST, seed thickness in mm; SW, hundred seed weight in grams; YLD, yield in grams.

Figure 4 illustrates the Spearman rank correlation coefficients between the ordinal traits (EPV and LP) and different agronomic traits, with significance levels indicated by asterisks. Spearman's correlation coefficient (*ρ*) ranges from −1 to +1 and measures the strength and direction of monotonic relationships between variables. The magnitude of the coefficients can be interpreted using the benchmark proposed by Jacob Cohen as small (|*ρ*| ≤ |0.10), medium (0.10 ≤ |*ρ*| ≤ 0.30), large (0.30 ≤ |*ρ*| < 0.50), and strong (|*ρ*| ≥ 0.50), providing an estimate of the effect size of the association.

Early plant vigor in both field (Figure 4a) and pot (Figure 4b) experiments showed a value from small to medium in strength of correlation with most of the quantitative traits, and most of the medium correlations showed statistically significant results. Medium positive correlation was observed across the two sets of experiments between EPV and SW (*ρ* = 0.16, *p* < 0.05), EPV and SD (*ρ* = 0.14 to 0.18, but not significant). It indicated that an increase in EPV resulted in a slight increase in seed size related traits. Conversely, medium negative relationships were detected between EPV and DF (*ρ* = −0.22 to −0.23, *p* < 0.01), EPV and SPP (*ρ* = −0.12 to 0.15, but not significant). These negative associations indicate that increases in EPV levels tend to correspond with slight reductions in these traits. However, the strength of correlation for DM, HI, PH, PPP, and YLD varied across the two experiments, suggesting that these traits are influenced by environmental conditions. Overall, the effect sizes for EPV suggest that while several relationships are statistically significant, their magnitudes are generally small, indicating a limited influence of EPV on most quantitative traits.

In contrast, LP exhibited stronger effect sizes and more consistent associations with the agronomic traits across the two sets of experiments. The strongest relationship was observed between LP and DM (*ρ* = 0.41 to 0.53, *p* < 0.001), representing a strong effect size, which indicates that higher LP levels are associated with longer maturity periods. Medium positive associations were also observed with SW (*ρ* = 0.21 to 0.24, *p* < 0.01), SD (*ρ* = 0.24 to 0.26, *p* < 0.001), and ST (*ρ* = 0.15 to 0.19, *p* < 0.05) across the two sets of experiments. Conversely, SPP (*ρ* = −0.27 to −0.35, *p* < 0.001) and PPP (*ρ* = −0.23 to −0.35, *p* < 0.01) exhibited medium to large negative correlations, indicating that increased leaf hairiness is associated with a decrease in these traits. Overall, the Spearman correlation analysis indicated that almost all relationships between the ordinal and continuous variables are medium to large in effect size, with most of them statistically significant. However, the stronger associations observed for LP compared with EPV suggest that LP may serve as a more influential trait in determining variation in certain agronomic characteristics specifically related to days to maturity, seed, and pod per plant, which may reveal that it has a significant role for trait evaluation and selection in breeding programs.

### 3.4 Effect Size Analysis of Polychotomous Variables on Agronomic Traits

**FIGURE 5:**
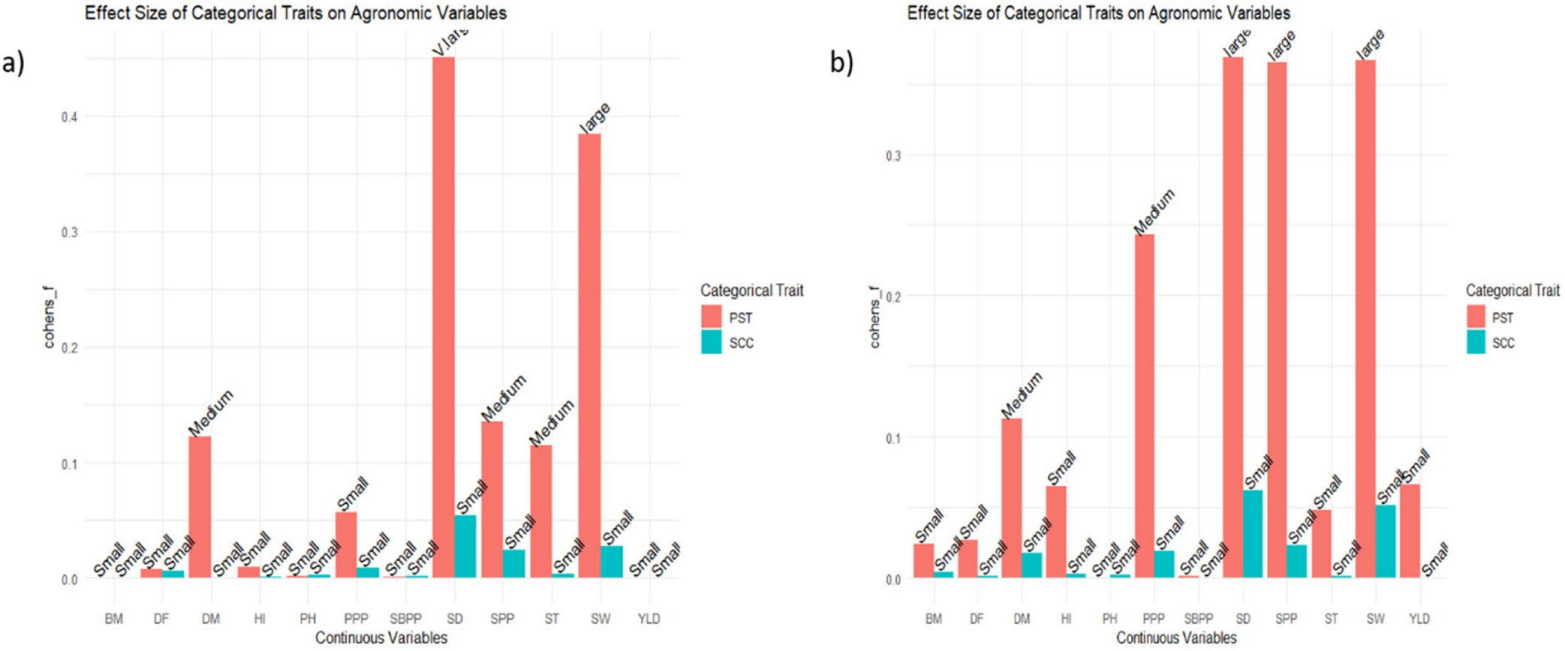
Comparison of Cohen's *f* effect sizes for seed coat color (SCC) and pattern of seed testa (PST) on agronomic traits. (a) Represent field experiment and (b) represent pot experiment and illustrate the effect size across different agronomic traits (*x*‐axis) and nominal variables (PST and SCC). The bars represent the magnitude of variance explained by group membership for each agronomic trait. Effect size benchmarks are indicated by horizontal lines (small = 0.10, medium = 0.25, large = 0.40) following (Cohen 1988) guidelines; BM, biomass yield in gram; DF, days to flowering; DM, days to maturity; HI, Harvest index; PPP, number of pods per plant; SBPP, number of secondary branches per plant; SD, seed diameter in mm; SPP, number of seeds per plant; ST, seed thickness in mm; SW, hundred seed weight in gram; YLD, yield in gram.

Figure 5a,b shows the effect sizes (Cohen's *f*) of two categorical traits (PST and SCC) on several agronomic traits with the field and pot experiments, respectively. Effect sizes were interpreted using the benchmarks proposed by Jacob Cohen: *f* = 0.10 (small), 0.25 (medium), and 0.40 (large).

For PST, most agronomic traits showed small to medium effect sizes, with a few traits exhibiting large effects. A large effect was observed for SD in the pot experiment (*f* = 0.37), while a very large effect was recorded in the field experiment (*f* = 0.45), suggesting that variation in PST categories strongly influenced seed diameter. Similarly, SW showed a large effect (*f* = 0.36 to 0.38). The effect size of PST on DM was medium across the two experiments, while its effect fluctuated on SPP from medium in the field experiment to large in the pot experiment. Similarly, the effect size of PST on PPP and ST varied from small to medium, indicating that environmental conditions may modulate the influence of this trait. In contrast, traits such as BM, DF, HI, and PH showed small or negligible effect sizes, suggesting that PST contributes little to the variation in these traits. For SCC, the effect sizes were generally small across all agronomic traits that showed a relatively weak influence of this categorical variable on the measured traits. Slightly higher effects were observed for SD, SW, and SPP, although these still remained within the small effect range. The other traits, BM, DF, DM, HI, PH, PPP, and YLD, showed negligible to small effects. This suggests that SCC has limited explanatory power for variation in these agronomic characteristics.

Overall, the results indicated that PST has a more pronounced influence on seed‐related traits particularly: seed diameter and seed weight. On the other hand, SCC showed minimal effects across the majority of traits. This pattern suggests that PST may be a more informative categorical factor for explaining variability in key agronomic characteristics, especially those related to seed morphology and productivity. Such findings may be useful for identifying traits that respond more strongly to categorical classifications and may therefore have greater relevance in trait evaluation or breeding programs.

### 3.5 Effect Size Analysis of Binary Variables on Agronomic Traits

**FIGURE 6:**
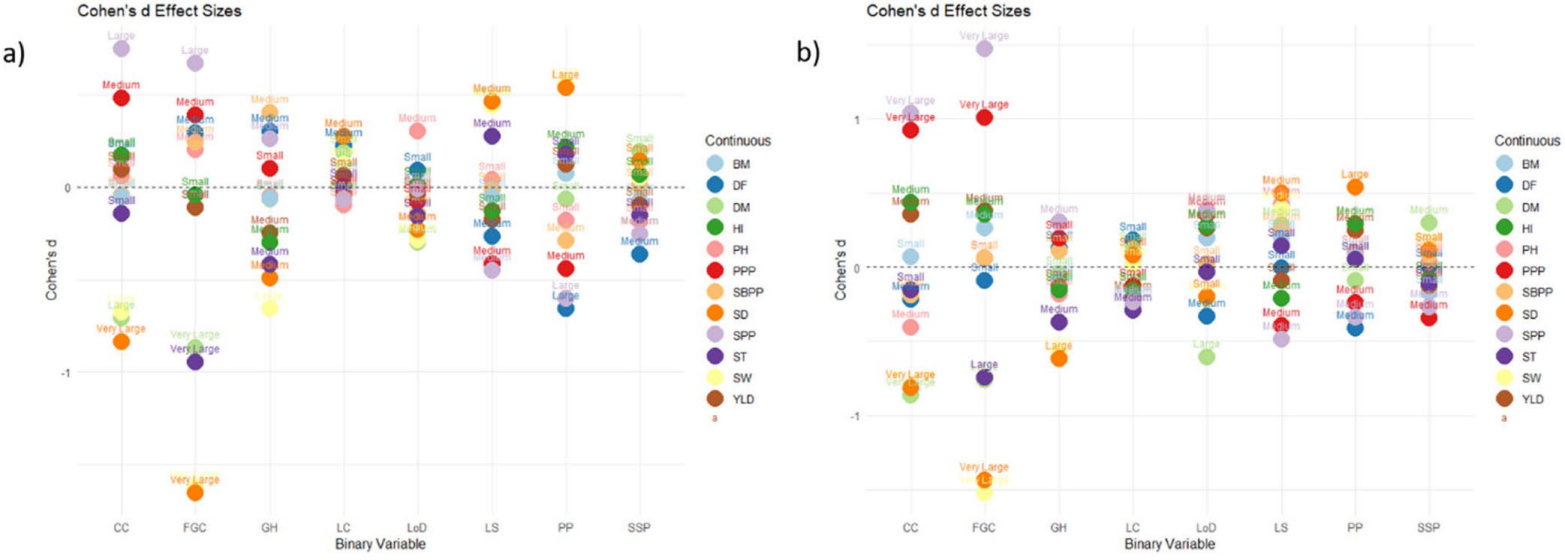
Cohen's *d* effect sizes for group differences across binary predictors and agronomic traits. (a) Represent field experiment and (b) represent pot experiment and illustrate the effect size (y‐axis) of binary variables on agronomic traits (*x*‐axis). Horizontal dashed line represents zero (no difference). Effect size benchmark (small = 0.20, medium = 0.50, large = 0.80) following (Cohen 1988); BM, biomass yield in gram; CC, cotyledon color; DF, days to flowering; DM, days to maturity; FGC, Flower ground color; GH, Growth habit; HI, Harvest index; LC, leaf color; LS, Leaf size; PP, pod pigmentation; PPP, number of pods per plant; SBPP, number of secondary branches per plant; SD, seed diameter in mm; SPP, number of seeds per plant; SSP, Seedling stem pigmentation; ST, seed thickness in mm; SW, hundred seed weight in gram; YLD, yield in gram.

The results of effect size analysis for the field and pot experiments are depicted in Figure 6a,b, respectively. The magnitude of effects is categorized as small, medium, large, and very large according to standard Cohen's *d* thresholds. The majority of comparisons showed small to medium effect sizes, indicating that most of these traits exert modest influences on the agronomic traits. These moderate magnitudes suggest that while differences between trait categories exist, their practical impact on the agronomic traits is generally limited. However, large and very large effect sizes were observed for specific trait combinations. Particularly, CC and FGC showed the largest effects across several agronomic traits. FGC showed very large negative effect sizes on SD, ST, and DM. It also showed medium to large positive effect on PH, SBPP, DF, PPP, and SPP. Similarly, CC demonstrated large and medium positive effect on SPP and PPP, respectively. It showed a negative effect size on SD, SW, and DM which is a strong differentiation between its groups. The orange cotyledon group tends to have higher SPP and PPP but lower SD, SW, and DM.

Leaf size showed a medium negative effect on several yield‐related traits. In contrast, it showed a medium positive effect on ST and SD, indicating that while increased LS may reduce reproductive output, it may still contribute to seed development. Pod pigmentation demonstrated medium to large negative effects on SBPP, PPP, SPP, and DF. However, PP had a medium positive effect on HI and a large positive effect on SD. These results suggest that although the presence of PP may negatively influence some yield components, it can enhance the efficiency of biomass partitioning and increase seed size. GH showed a large negative effect on SW while it has a medium negative effect on SD, ST, HI, and PPP. On the other hand, it showed a medium positive effect on SBPP, DF, and SPP. Other traits, such as LC, LoD, and SSP, generally showed small to moderate effect sizes.

The presence of both positive and negative effect sizes showed the direction of the group differences, where positive values indicate higher average values of the agronomic traits in one category of the binary trait and negative values indicate higher average value in the alternative category (see Section 3.5 for more clarity). Overall, the effect size analysis revealed that many trait combinations showed modest differences while some binary traits, particularly FGC and CC, showed large effects on several agronomic traits, highlighting their potential importance in influencing agronomic performance.

### 3.6 Principal Component Analyses of Qualitative and Quantitative Traits

**FIGURE 7:**
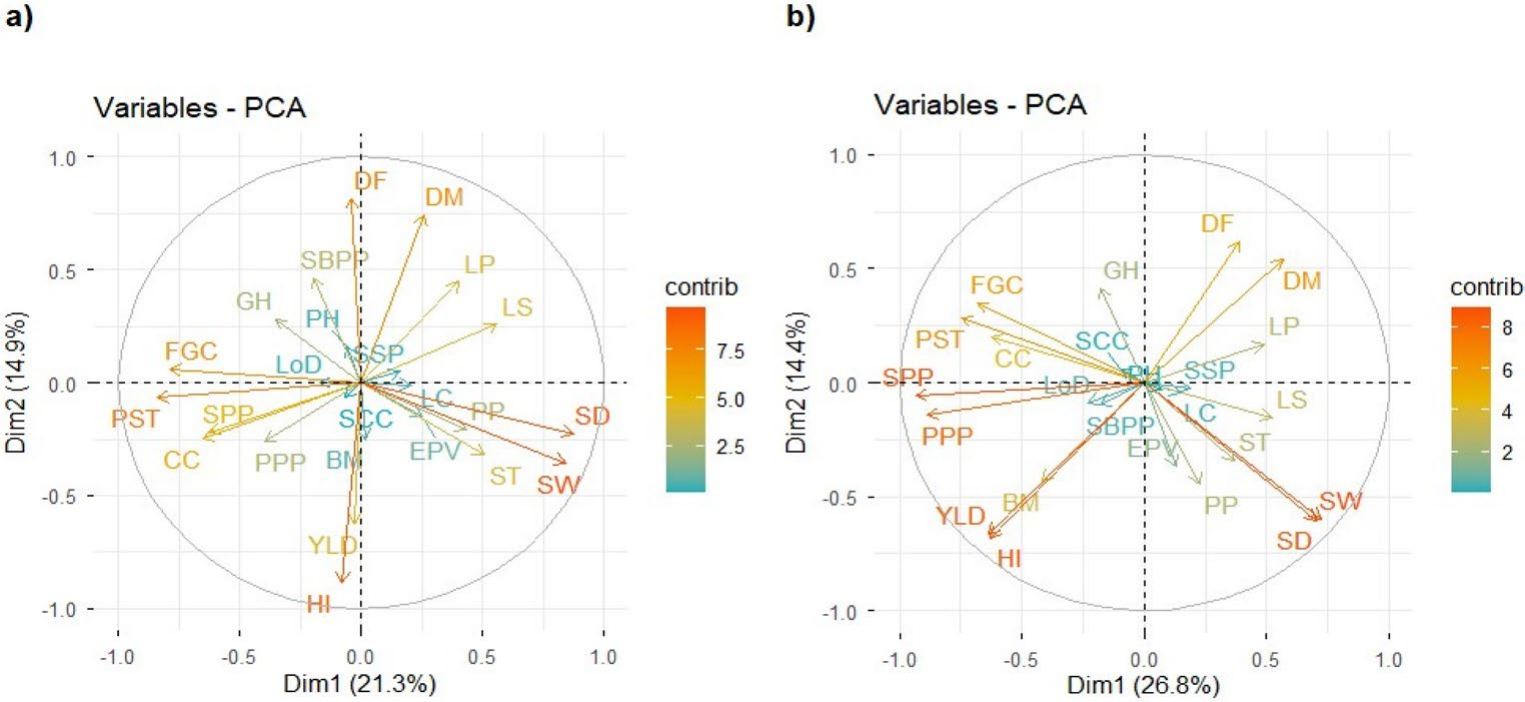
Principal component analysis explaining the correlation and contribution of different agronomic and qualitative traits of lentil germplasm, (a) Represents field experiment and (b) Represents pot experiment; BM, biomass yield in gram; CC, cotyledon color; DF, days to flowering; DM, days to maturity; EPV, early plant vigor; FGC, Flower ground color; GH, Growth habit; HI, Harvest index; LC, leaf color; LP, Leaf pubescence; LS, Leaf size; PPP, number of pods per plant; PP, pod pigmentation; PST, Pattern of seed testa; SBPP, number of secondary branches per plant; SCC, Seed coat color; SD, seed diameter in mm; SPP, number of seeds per plant; SSP, Seedling stem pigmentation; ST, seed thickness in mm; SW, hundred seed weight in gram; YLD, yield in gram.

Principal Component Analysis (PCA) biplot was used to define and understand the most important traits. It also shows correlation between either germplasm or between traits in a two‐dimensional plane. The PCA biplot for the field and pot experiments illustrated the contribution of individual traits to the observed variation and the relationships among these traits (Figure 7a,b, respectively). The distribution and orientation of trait vectors in the biplot indicate the relative importance of each trait in explaining phenotypic variability, as well as the degree of association among traits across the evaluated germplasm. The first five principal components explained 62.41% and 63.07% of the total variance in the field and pot experiments, respectively, with most of the individual trait variations accounted for by PC1, PC4, and PC5 (Table S3). While PC1 captured the largest fraction of variation, PC4 and PC5 also contributed substantial variation for EPV, LC, LP, LoD, GH, SSP, and SCC, indicating that both primary and secondary sources of variation are important in explaining differences among germplasm. The first two principal components (PC1 and PC2) explained 36.2% and 41.2% of the total variance in the field and pot experiments, respectively (Table S3). The most important qualitative characters contributing large variation to PC1 were FGC, PST, CC, LP, LS, PP, and GH. They were also found to be important characters for distinguishing the different populations of lentil germplasm. On the other hand, YLD, HI, SD, SW, PPP, and SPP were the most important agronomic traits that described the variation of lentil germplasm with the first two PCs. However, LoD, SSP, LC, EPV, and SCC were not significant in determining the variation of the lentil germplasm.

The PCA biplot also showed the association between both qualitative and agronomic traits. SW, SD, ST, and PP were found in close proximity to each other in the same direction, indicative that seed size related traits were positively associated with pod pigmentation. However, the seed size related traits were found negatively associated with FGC, PST, and CC as they were found in opposite directions on the PC plane. Days to flowering, DM, LP, and LS were grouped together and found in the same direction on the principal component (PC) plane and hence the phenology of lentil positively associated with leaf pubescence and leaf size. Pattern of seed testa, FGC, CC, SPP, and PPP were positioned in the same direction on the PC plane, indicating a close relationship among them. The reproductive traits (podding and seed development) of lentil have a significant and positive association with the pattern of seed testa, cotyledon color, and flower ground color. On the other hand, reproductive traits of lentil were found negatively associated with leaf pubescence and leaf size.

**FIGURE 8:**
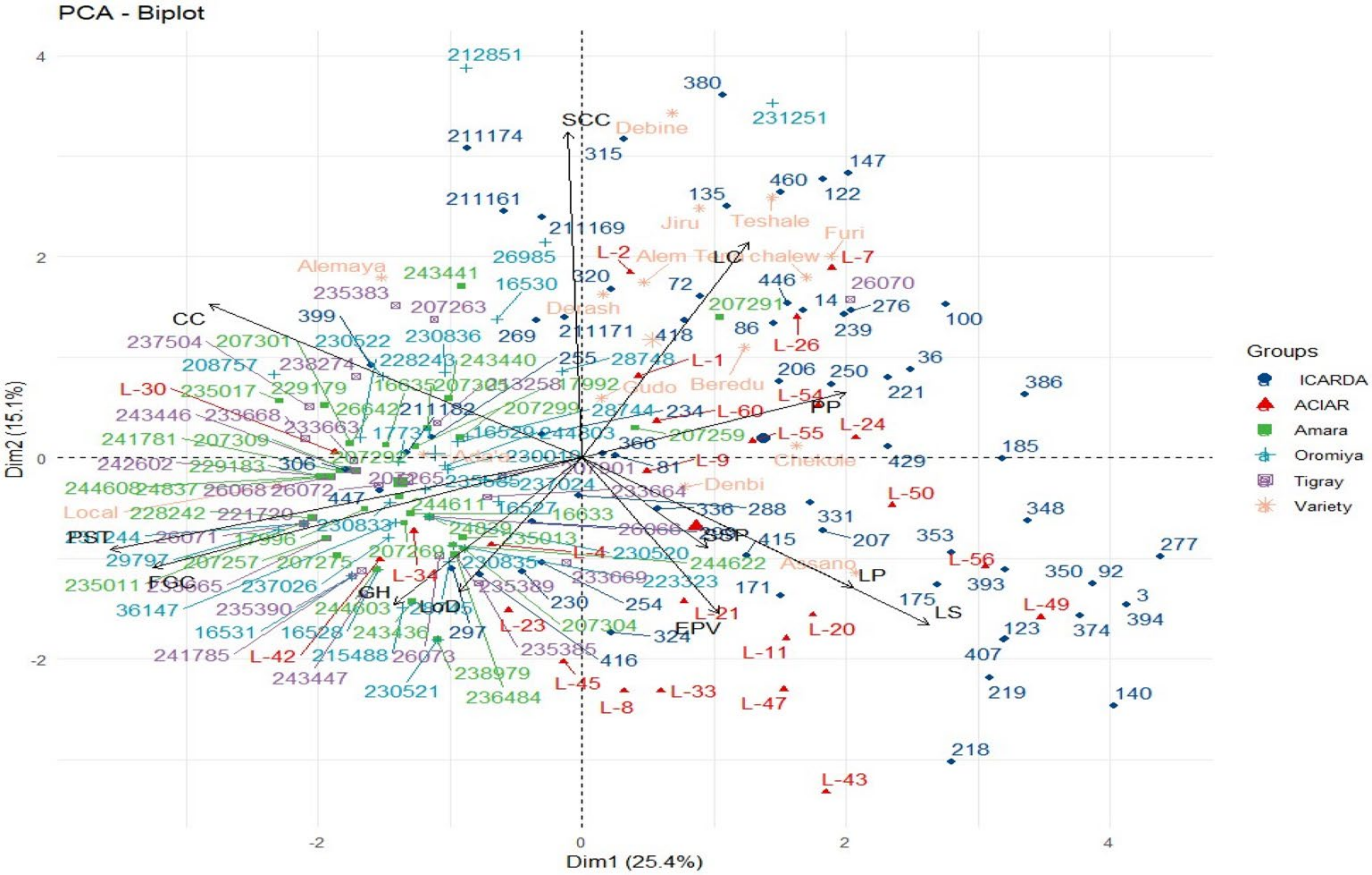
Distribution of germplasm on principal component (PC) plane based on lentil qualitative characters. CC, cotyledon color; EPV, early plant vigor; FGC, Flower ground color; GH, Growth habit; LC, leaf color; LP, Leaf pubescence; LS, Leaf size; PP, pod pigmentation; PST, Pattern of seed testa; SCC, Seed coat color; SSP, Seedling stem pigmentation.

PCA biplot explained 40.5% of the total variation in the first two dimensions for germplasm is depicted in Figure 8. It illustrated the diversity present within the germplasm and highlights the relationship between qualitative traits and the geographic origin of the germplasm. Accessions from Oromia, Amhara, and Tigray were primarily associated with PST, FGC, CC, LoD, and GH, and clustered together around the center. They are mainly characterized by a complex pattern of seed testa, pink flower color, and orange cotyledon color, and were semi‐erect type and susceptible to lodging. Variation along the negative side of the first principal component was largely explained by CC, PST, and FGC, which separated lentil landraces from exotic ones. The PC plot also showed that there is negligible variation among landraces (Amhara, Oromiya, and Tigray) in terms of their qualitative traits. ACIAR and ICARDA lines were distinguished by variation in LS, LC, PP, LP, and EPV, and can be described as medium to large leaf size, green leaf color, pigmented pod, and medium to dense leaf pubescence and with vigorous seedling. In addition, ACIAR and ICARDA materials showed the widest distribution across the plot, reflecting their broad genetic diversity.

### 3.7 Comparing Group Mean From Welch's ANOVA and Welch's t Test

Mean values of agronomic traits across categories of qualitative traits for both field and pot experiments are presented in Table 3. Correlation and effect size analyses identified PST, CC, FGC, LP, PP, LS, and GH as key qualitative traits in lentil due to their substantial effects on several agronomic traits. Accordingly, considerable variation in agronomic and yield‐related traits was observed among the evaluated germplasm across groups of these categorical variables. The magnitude of variation in agronomic characters differed across categorical traits, which indicates potential associations between qualitative characteristics and yield components.

**TABLE 3:** Mean value of each agronomic trait across different categorical variables (characters) for pot and field experiment.

Traits	Exp.	Categories	DF	DM	SBPP	PPP	SPP	YLD	SW	HI	SD	ST
GH	Pot	Sem Erect	63.17^a^	98.09	3.54	34.77	48.58^a^	1.44	3.09^b^	24.25	4.59^b^	2.43^b^
		Erect	61.03^b^	96.4	3.36	33.24	44.66^b^	1.5	3.54^a^	25.29	4.88^a^	2.48^a^
	Field	Sem Erect	65.55	108.98	4.39^a^	58.01	92.06^a^	63.05^b^	2.69^b^	30.24^b^	4.55^b^	2.42^b^
		Erect	63.91	108.42	3.66^b^	56.72	86.50^b^	71.74^a^	3.17^a^	32.61^a^	4.83^a^	2.48^a^
LP	Pot	Absent	60.09^b^	93.64^b^	3.3	37.16^a^	50.71^a^	1.51^a^	3.03^b^	25.57^a^	4.57^b^	2.43^b^
		Slight	60.42^b^	95.17^b^	3.47	34.99^a^	48.34^a^	1.55^a^	3.36^ab^	25.85^a^	4.77^a^	2.45^ab^
		Medium	65.95^a^	103.27^a^	3.49	29.21^b^	39.12^b^	1.33^b^	3.64^a^	22.62^b^	4.91^a^	2.49^a^
	Field	Absent	63.71	105.75^b^	4.02	61.46^a^	97.48^a^	70.75^a^	2.72^b^	32.88^a^	4.51^b^	2.42
		Slight	63.69	106.63^b^	3.77	58.53^a^	92.48^a^	71.39^a^	2.99^ab^	32.83^a^	4.72^ab^	2.47
		Medium	66.81	114.46^a^	4.22	51.53^b^	75.96^b^	60.54^b^	3.15^a^	28.57^b^	4.87^a^	2.47
LS	Pot	Small	60.76	95.45^b^	3.29	35.71^a^	49.55^a^	1.5	3.09^b^	25.51	4.6^b^	2.45
		Medium	62.73	98.27^a^	3.53	32.6^b^	44.02^b^	1.46	3.53^a^	24.4	4.87^a^	2.46
	Field	Small	63.72	106.06^b^	3.79	60.17^a^	95.43^a^	70.48	2.75^b^	32.86^a^	4.55^b^	2.45
		Medium	65.2	110.47^a^	4.08	55.22^b^	84.32^b^	66.47	3.12^a^	30.76^b^	4.82^a^	2.46
FGC	Pot	White	62.37	99.28^a^	3.34	28.57^b^	35.62^b^	1.39^b^	4.10^a^	23.75^b^	5.19^a^	2.52^a^
		Purple	61.7	96.02^b^	3.48	36.53^a^	51.63^a^	1.52^a^	2.98^b^	25.41^a^	4.55^b^	2.42^b^
	Field	White	64.44	111.03^a^	3.62^b^	52.88^b^	75.94^b^	69.56	3.61^a^	31.32	5.18^a^	2.54^a^
		Purple	64.67	107.47^b^	4.13^a^	59.44^a^	95.58^a^	67.40	2.64^b^	31.77	4.47^b^	2.41^b^
PP	Pot	Absent	62.52	97.59	3.34	34.46	47.81^a^	1.45	3.15^b^	24.47	4.64^b^	2.46
		Present	60.75	96.17	3.62	32.74	43.32^b^	1.52	3.74^a^	25.6	5.00^a^	2.45
	Field	Absent	65.68^a^	109.32	4.22^a^	59.40^a^	94.56^a^	67.56	2.79^b^	31.01	4.59^b^	2.45
		Present	62.45^b^	107.36	3.47^b^	53.07^b^	78.12^b^	69.21	3.31^a^	32.82	4.94^a^	2.47
SCC	Pot	Green	63.92^a^	101.7^a^	3.49	28.91^b^	36.94^b^	1.38^b^	3.97^a^	23.47^b^	5.14^a^	2.49^a^
		Gray	63.21^ab^	98.81^a^	3.48	32.13^b^	42.40^b^	1.39^b^	3.43^b^	24.04^ab^	4.78^b^	2.48^a^
		Brown	60.35^b^	93.85^b^	3.39	37.38^a^	53.05^a^	1.55^a^	2.96^c^	25.92^a^	4.53^c^	2.43^b^
	Field	Green	65.08	112.28^a^	3.93	53.11^b^	75.68^b^	67.35	3.47^a^	30.52	5.12^a^	2.50^a^
		Gray	67.06	113.37^a^	4.35	57.5^ab^	89.13^a^	70.37	3.13^b^	30.85	4.76^b^	2.52^a^
		Brown	63.62	105.16^b^	3.88	59.71^a^	97.21^a^	67.97	2.62^c^	32.5	4.44^c^	2.41^b^
PST	Pot	Absent	62.55^b^	99.61^b^	3.46	28.01^b^	35.29^b^	1.37^b^	4.09^a^	23.69^b^	5.20^a^	2.51^a^
		Spotted	67.91^a^	103.96^a^	3.53	35.05^a^	46.98^a^	1.44^ab^	3.13^b^	23.76^ab^	4.67^b^	2.39^b^
		Complex	60.39^b^	94.31^c^	3.4	37.08^a^	52.58^a^	1.54^a^	2.97^b^	25.75^a^	4.52^b^	2.44^b^
	Field	Absent	64.84^b^	112.02^a^	3.83^b^	51.10^b^	73.74^b^	66.87	3.62^a^	31.14	5.20^a^	2.53^a^
		Spotted	69.82^a^	114.56^a^	4.67^a^	62.60^a^	92.96^b^	73.3	2.8^b^	29.36	4.58^b^	2.41^b^
		Complex	63.43^b^	105.55^b^	3.9^ab^	59.81^a^	97.31^a^	67.84	2.62^b^	32.35	4.45^b^	2.42^b^
CC	Pot	Yellow	66.12^a^	103.15^a^	3.64	25.97^b^	32.53^b^	1.34^b^	4.42^a^	22.74^b^	5.40^a^	2.48
		Orange	61.08^b^	95.90^b^	3.39	35.46^a^	49.05^a^	1.50^a^	3.14^b^	25.28^a^	4.63^b^	2.45
	Field	Yellow	67.09^a^	114.38^a^	4.24	50.08^b^	67.96^b^	63.72	3.77^a^	28.37^b^	5.34^a^	2.48
		Orange	64.09^b^	107.51^b^	3.91	58.69^a^	93.32^a^	69.00	2.81^b^	32.27^a^	4.58^b^	2.45

*Note:* Superscript letters indicate significance level at *p* ≤ 0.05.

Abbreviations: CC, cotyledon color; DF, days to flowering; DM, days to maturity; FGC, Flower ground color; GH, Growth habit; LP, Leaf pubescence; LS, Leaf size; PP, pod pigmentation; PPP, number of pods per plant; PST, Pattern of seed testa; SBPP, number of secondary branches per plant; SCC, Seed coat color; SD, seed diameter in mm; SPP, number of seeds per plant; ST, seed thickness in mm; SW, hundred seed weight in gram; YLD, yield in gram.

Days to maturity showed noticeable variation among groups of categorical traits. In the field experiment, germplasm without leaf pubescence matured earlier (105.75 days) than those with medium leaf pubescence (114.46 days). Germplasm with small leaves matured earlier (106.06 days) than those with medium leaves (110.47 days). On the other hand, lentils with white flowers, non‐patterned seeds, and orange cotyledons took longer to mature than those with purple flowers, patterned seeds, and yellow cotyledons across both experiments, indicating that lighter‐colored flower and seed traits are associated with prolonged maturity.

Pod per plant, an important yield component trait, varied among groups of leaf‐related traits and flower colors. In the field experiment, the mean PPP ranged from 51.53 for medium to 61.46 for germplasm without leaf pubescence, from 55.22 for medium to 60.17 for small leaf size, and from 52.88 for purple to 59.44 for white flower ground color. A similar trend was also observed for the two leaf‐related traits in the pot experiment. Variation was also observed for pod pigmentation, seed testa pattern, and cotyledon color. Across both experiments, lentil germplasm with non‐pigmented pods, more patterned seed testa, and orange cotyledons exhibited higher pod load. Seed per plant, another closely related yield component, also showed variation across categorical traits. Higher mean SPP values were observed in germplasm with semi‐erect growth habit, non‐pubescence leaf, and small leaf size. Similarly, germplasm that had purple flower color, non‐pigmented pod, complex seed testa pattern, and orange cotyledon color produced higher SPP values in the field experiment, with a similar trend also recorded in the pot experiment.

Seed yield was significantly affected only by GH and LP in field experiments, with greater seed yield recorded for erected growth type and with slight leaf pubescence. Erected lentil types yielded 8.69 g more than semi‐erect types. Yield was considerably lower with medium‐density leaf pubescence (60.54 g) as compared with slight‐density leaf pubescence (71.39 g). In the pot experiment, yield was significantly affected by LP, FGC, PST, and CC. Consistent with the field experiment, lentils with slight leaf pubescence produced the highest yield (1.55 g), while those with medium leaf pubescence recorded the lowest yield (1.33 g). Lentils with purple flower color produced on average 0.13 g higher yield than those with white flowers in the pot experiment; however, the variation was not significant in the field experiment. Lentil germplasm with complex pattern of seed testa had higher yield than those without and those with spotted seed testa in the pot experiment, while there is no significant variation in the field experiment. The orange seed coat color showed better seed yield in both types of experiments, and in general darker and more pigmented seeded lentil germplasm showed higher yield, while lentils with hairy leaves showed lower yield.

Growth habit showed a significant effect on seed weight, seed diameter, and seed thickness. The erect type showed superior seed size‐related traits in both sets of experiments. Leaf pubescence showed a significant effect on seed weight, seed diameter, and seed thickness. The medium leaf pubescence showed better seed size‐related traits than absent and slight leaf pubescence across the two experiments. Leaf size also showed a significant effect on seed weight and diameter with both field and pot experiments, and superior seed weight and diameter were recorded for medium leaf size. Flower ground color also showed a significant effect on seed weight, diameter, and thickness with both field and pot experiments, and better seed weight (3.61 g), seed diameter (5.18 mm), and thickness (2.54 mm) were recorded for white flower in the field experiment. A similar trend was observed in the pot experiment, where white‐flowered accessions showed the highest seed weight (4.10 g), seed diameter (5.19 mm), and seed thickness (2.52 mm). Pod pigmentation showed a significant effect on seed weight and diameter with both field and pot experiments. Pigmented pod exceeded the non‐pigmented pod by 0.52 g for seed weight and by 0.35 mm for seed diameter in the field and pot experiments. These results suggest that variation in key qualitative traits can influence seed development and size, highlighting their potential importance in the selection of desirable traits for lentil improvement.

Pattern of seed testa also showed a significant effect on seed size related traits. Highest seed weight, diameter, and thickness were recorded in germplasm with non‐patterned and spotted seed testa patterns in both field and pot experiments. Similarly, significant variation in seed weight and diameter was observed in relation to CC across both experiments, with yellow‐cotyledon germplasm showing higher seed weight and seed diameter. These results suggest that variation in PST and CC is associated with differences in seed development, with certain color types showing a stronger influence on seed size traits. Overall, several qualitative traits in lentil were found to significantly influence the agronomic performance of the crop.

## 4 Discussion

The goodness of fit test showed that all qualitative characters except LoD were significantly deviated from the expected frequency distribution probably due to human interference through selection or environmental effect on the population as described by Falconer (1989). Sharma et al. (2022) and Singh et al. (2018) also reported that all their recorded qualitative characters exhibited significant variation among their tested genotypes. The heterogeneity of these characters is important for tailoring varieties that meet farmers' needs (Aktar et al. 2024). The most frequent category of each qualitative trait was assessed in this study. The evaluated lentil germplasm was predominantly characterized by good plant vigor, presence of stem pigmentation, erect growth habit, green leaf color, slight leaf pubescence, medium leaf size, and purple flower color, absence of pod pigmentation, brown seed coat color, complex seed testa pattern, and orange cotyledon color. A similar result was reported by Tripathi et al. (2021) though they reported that semi erect growth habit was prominent.

Principal component analysis (PCA) technique, which simultaneously analyses multiple measurements on individuals is widely used in the analysis of genetic diversity (Mohanlal et al. 2023). PCA can be used to reveal similarities among variables, distinguish germplasm based on these variables, and assess the importance and contribution of the variables to the total variance (Ajaykumar et al. 2023; Mohanlal et al. 2023). In the current study, PCA showed that FGC, PST, CC, LP, LS, PP and GH were the most important characters in terms of the total variation of lentil germplasm obtained from different sources and hence contributing to various degree of adaption across environments. Similarly, a principal component analysis by Khatun et al. (2022) showed that Seed property, flower color and leaf related traits were an important qualitative characters in country bean. These characters were linked to adaptive physiological or structural traits that help the plant cope with stress faced across different environments (Pshenichnikova et al. 2019). Besides the qualitative traits, the PCA showed DF, DM, YLD, HI, SD, SW, PPP, SPP contributed greatly towards the variation observed across the lentil germplasm. These are economic traits of lentil, as noted by Sharma (2009). Studying the association of these economic traits with that of qualitative characters of lentil help breeders to identify morphological indicators of high performing genotypes and to understand the visible difference related to adaptation (Delcoco et al. 2022).

Association between traits and source of origin for the germplasm provides important understanding of these traits in a way that should help with adapting the preferred traits by producers and consumers of that specific environment (Khazaei et al. 2018). This is because qualitative characters are directly linked with agronomic performance, adaptation, and stress tolerance (Elessawy et al. 2023). The PCA indicated that landraces from Oromiya, Amhara, and Tigray were primarily associated or clustered with PST, FGC, CC, LoD, and GH. These landraces were characterized by a complex seed testa pattern, purple flowers, orange cotyledons, and a semi‐erect growth habit with lodging tendency. Najjar et al. (2025) reported that these types of characteristics are highly preferred by farmers and consumers in Ethiopia and cope with stress conditions. Orange cotyledon color was the dominant characteristic of landraces, which also influences the seed coat color of lentils, as previously reported by Bekele et al. (2023). ACIAR and ICARDA germplasm showed broad genetic diversity defined by medium to large leaf size, green to light leaf color, pigmented pod to absent, medium to dense leaf, white to purple flower, yellow to orange cotyledon, lighter than darker seed coat, and somewhat vigorous seedlings. Similar observations were also reported in the previous study by Kefelegn et al. (2024).

Research on determining the association within qualitative, and between qualitative and agronomic characters is very limited. However, Mashilo et al. (2016) showed that qualitative traits could be associated among each other and also with yield and yield‐related traits in bottle gourd landraces. They reported that the estimation of correlation among qualitative characters may guide targeted breeding of the crop through considering these valuable traits. However, correlations among qualitative, and between qualitative and agronomic traits have not been reported in lentil not only in Ethiopia but also worldwide; however, Khatun et al. (2022) reported that there was a strong relationship among flower color, seed characters, and leaf properties in country bean. In the current study, Cramer's V analysis showed several meaningful associations among the studied morphological traits. Strong associations were observed among PST, SCC, and FGC, indicating that these traits are closely related and may share common genetic or physiological mechanisms governing their expression, which align with the study report from country bean (Khatun et al. 2022). This strong relationship suggests that variation in one of these traits is likely accompanied by variation in the others, reflecting a coordinated expression of seed and flower characters. Cotyledon color also exhibited moderate associations with the two seed coat traits (SCC and PST), implying a potential genetic or developmental linkage between cotyledon pigmentation and seed coat characteristics. In addition, the two seed‐related traits showed moderate associations with leaf‐related traits, including LC, LP, and LS, as well as with PP. Similarly, Khatun et al. (2022) also showed that CC associated with seed color and also with leaf color in country bean. These relationships suggest a partial linkage between seed‐related traits and vegetative as well as pod pigmentation characteristics, possibly due to shared developmental pathways or genetic features. Furthermore, LP demonstrated a moderate association with LS and FGC, indicating that leaf characters may also be partially connected with flower characters. Overall, this study highlights the interconnected nature of seed, leaf, flower, and pod traits, as also reported in country bean by Khatun et al. (2022).

Agronomic traits also showed various degrees of connection with qualitative traits (categorical variables). Vegetative related traits (SBPP and BM), YLD and HI showed weak and non‐significant relationships with most of the categorical variables. However, phenological traits (DF and DM), seed size–related traits (SW, ST, and SD), and some yield‐related traits (PPP and SPP) showed moderate to strong associations with these qualitative characters, suggesting that qualitative traits can serve as indirect selection markers to improve agronomic performance in lentil.

Days to maturity was positively associated with LP, with germplasm exhibiting medium leaf pubescence showing delayed maturity. This indicates that as the level of leaf pubescence increased, the number of days to maturity also tended to increase. There was about a 10‐day difference in maturity between non‐hairy and moderately hairy lentil germplasm. Gorim and Vandenberg (2017) reported that leaf pubescence can prolong the phenology of lentil through preventing overheating and water stress. Based on Cohen's *f* calculation, patterned seed showed a medium effect size on days to flowering and maturity, and the mean comparison showed that there is about 8 day's interval between non patterned and complex patterned seeded lentil germplasm. Rapid depletion of soil moisture during flowering and maturity accelerates phenological development in lentil, leading to earlier flowering and maturation. This response may be associated with enhanced flavonoid metabolism in darker and patterned seeds, as reported by Butu et al. (2014) and Jiang et al. (2024). Other studies have also reported that higher flavonoid accumulation is commonly observed in darker‐colored crop seeds (Iqbal et al. 2025; Roy et al. 2025; Zhao et al. 2025). Flavonoids can interact with and modulate the accumulation and signaling of Abscisic acid (ABA), a key regulator of plant responses to water deficit. Increased ABA levels, together with flavonoid‐mediated stress responses, are known to promote early maturation and stress escape mechanisms (Nguyen et al. 2013; Nakabayashi et al. 2014; Wan et al. 2016; Yang et al. 2021; Muhammad et al. 2022). Another previous study reported that seed pigmentation played a key role in the adaptive strategies employed by plants in response to changing environmental conditions (Jaiswal and Dakora 2024). Furthermore, the Cohen's *d* analysis indicated that CC and FGC had a large negative effect on the crop phenology, and the Welch's *t*‐test further revealed that germplasm possessing orange cotyledons and purple flowers tended to mature earlier than those with alternative‐colored traits, probably with the same reason mentioned by Nguyen et al. (2013) and Muhammad et al. (2022).

Pattern of seed testa showed medium to large effect size (Cohen's *f*) on PPP and SPP, suggesting that variation in seed testa pattern contributes substantially to the differences observed in these traits. Selection based on PST could lead to noticeable differences in PPP and SPP. Similarly, the Cohen's *d* analysis showed a positive association between yield‐related traits (PPP and SPP) and the categorical variables FGC and CC, indicating a potential linkage between these agronomic traits and the categorical variables. Better number of pod and seed per plant was obtained in more darker or patterned seed, purple flower and orange cotyledon lentil germplasm. Related study in flax showed that seed color associates with yield related traits like number of capsule, number of seed per capsule and yield (Abtahi et al. 2022). As previously mentioned, the flavonoid driven anthocyanin production in these traits increase early flowering which probably gives the crop more growing and reproductive period under terminal moisture stress areas. On the other hand, the negative association of these yield related traits with leaf pubescence showed that leaf without leaf hair responded for better number of pod and seed per plant. A similar result was reported by Bahraminejad et al. (2011) where they mentioned that leaf without trichome responded for better yield related traits in cumin. Although the effect size varied from small to moderate across the two experiment, the Welch's ANOVA test showed that the mean seed yield per plant differed significantly among the categories of FGC and CC. Lentil germplasm with purple flowers and yellow cotyledons tend to produce higher yields probably due to pigmented traits which directly linked to enhanced stress resilience, better reproductive success, and efficient seed development, either through metabolic allocation or protective physiological mechanisms as described in previous studies (Muhammad et al. 2022; Wang et al. 2022). On the other hand, mean yield differed significantly among LP groups, and the corresponding effect size was categorized as medium. The mean yield was lower in lentil germplasm with medium leaf pubescence than the alternatives, likely due to reduced absorption of photosynthetically active radiation, as previously reported by Ehleringer et al. (1976), Bahraminejad et al. (2011) and Hussain et al. (2022).

The relatively large negative Cohen's *d* values observed for FGC and CC on seed size related traits indicate a strong association between these traits. Germplasm with purple flowers and orange cotyledons showed smaller seed size than their alternative trait expressions. Anthocyanin represents a kind of flavonoid compound as primary pigments (color) of many plants, and they play essential roles in plant defense responses to abiotic stresses and alter the synthetic pathway in plant tissue development (Kitamura et al. 2004; Wang et al. 2022). Hence, probably orange cotyledon colored and purple‐flowered germplasm have higher anthocyanin synthesis, indicating higher activity of the phenyl‐propanoid pathway, which competes with carbohydrate storage pathways. This competition may lead to smaller seed size or lower seed weight in pigmented germplasm either with their flower or seed cotyledon. Kitamura et al. (2004) mentioned that cross talk among the various pathways must play a major role in the control of seed development. On the other hand, white‐flowered and yellow‐cotyledon germplasm, which are considered lightly pigmented and likely exhibit lower anthocyanin activity, may allocate more resources to storage tissue formation, resulting in larger seeds. Another important observation of the current study was the large Cohen's *f* value for PST and its strong effect on seed size of lentils. Seed size related traits were greatly diminished with heavily pigmented or patterned seeds. Two possible scenarios may explain this phenomenon. The first, as described by Jaimie et al. (2005), suggests that strong competition exists among flavonoid and anthocyanin biosynthesis pathways and the pathways responsible for carbohydrate storage, protein accumulation, and amino acid synthesis during seed development, which may result in reduced seed size. The second scenario, heavily pigmented seeds often have thicker seed coats and higher phenolic content which in turn could reduce seed expansion during development and shorten maturation and restrict nutrient flow to the embryo, potentially lowering seed size (Miller et al. 1999).

This research finding verified that large seeded germplasm was associated with yellow cotyledon, while small seeded germplasm was associated with orange cotyledon color. Vijay et al. (2014) noted that macrosperma germplasm has yellow cotyledons while microsperma germplasm had orange cotyledons. The cotyledon color is governed by carotenoid and flavonoid biosynthesis pathways that are directly involved with seed developmental metabolism (Kitamura et al. 2004). Antioxidant or phenolic metabolism increases in pigmentation (orange cotyledon, patterned seed and purple flower) that probably involves a trade‐off with seed enlargement due to resource allocation challenges (Brend et al. 2012; Kitamura et al. 2004). In a stress environment, the biochemical composition of the seed changes, and accumulation of storage proteins, amino acids and carbohydrates declines in the seed (Singh et al. 2022). Yellow cotyledons generally have lower phenolic and tannin contents, which may promote greater accumulation of storage proteins and carbohydrates, leading to larger seeds or higher seed weight (Brend et al. 2012). In general, this study demonstrates that certain qualitative traits play a significant role and show strong interactions with certain agronomic traits, likely due to their close association with specific metabolic pathways and the production of particular secondary metabolites, as discussed herein.

## 5 Conclusions

Pigmentation in the reproductive part (purple flower and pigmented pod) and seed characters (complex patterns, brown or darker seed coats and yellow cotyledons) were found to be the most important characters in determining the agronomic performance and adaptation of lentil. Besides, qualitative characters were found important to determine the economic value of the crop such as seed size, seed color, and yield. Despite their low diversity, landraces were found to be more pigmented in their flowers, seed coats, and cotyledons, which helps them perform better and also preferred more in moisture deficit areas. In contrast, the wide genetic diversity present in the ICARDA and ACIAR lentil germplasm provides an excellent opportunity for breeders to explore desirable traits, particularly those associated with yield and large‐seeded genotypes. In general, qualitative traits were found to be highly important for identifying economically valuable agronomic traits within diverse lentil germplasm, either directly or indirectly, when making selections in lentil breeding programs. Therefore, breeders should consider qualitative characters and their interactions with agronomic traits in lentil to facilitate the identification and selection of the most desirable or economically important genotypes from diverse germplasm groups. Overall, this study showed that seed‐related characters, flower color, and leaf‐related characters are interrelated and associated with several agronomic traits, and may help to define the objectives of lentil breeding programs and provide a strong foundation for further studies on trait linkage, association mapping, and the underlying molecular mechanisms controlling these traits.

## Funding

The publication and the project work were supported by Australian Centre for International Agricultural Research (ACIAR), Amhara Agricultural Research Institute and Addis Ababa Science and Technology University.

## Conflicts of Interest

The authors declare no conflicts of interest.

## Supporting information


**Figure S1:** Residual fitted model for Days to flowering.
**Figure S2:** Residual fitted model for Days to maturity.
**Figure S3:** Residual fitted model for Plant height.
**Figure S4:** Residual fitted model for Secondary branch per plant.
**Figure S5:** Residual fitted model for Pod per plant.
**Figure S6:** Residual fitted model for Seed per plants.
**Figure S7:** Residual fitted model for Yield.
**Figure S8:** Residual fitted model for Seed weight.
**Figure S9:** Residual fitted model for Seed diameter.
**Figure S10:** Residual fitted model for Seed thickness.

**Table S1:** List of planting material (germplasm) by their source of origin.
**Table S2:** Contingency table of categorical variables.
**Table S3:** The first five principal components for both experiments.

**Data S1:** Data containing accession name, pedigree, source or population and some important qualitative characters of each individual samples.

## Data Availability

The data supporting the results of this study are available in the Supporting Information of this article.
