# Supplementary material for: Association of Qualitative Characters With Agronomic Traits, and Their Breeding Importance in Lentil (Lens culinaris Medikus)
Source: Plant Environ Interact. 2026 May 13;7(3):e70162. doi: 10.1002/pei3.70162 (PMC13172294; doi:10.1002/pei3.70162)
Supplement: Supplementary file 1 — Figure S1: Residual fitted model for Days to flowering. Figure S2: Residual fitted model for Days to maturity. Figure S3: Residual fitted model for Plant height. Figure S4: Residual fitted model for Secondary branch per plant. Figure S5: Residual fitted model for Pod per plant. Figure S6: Residual fitted model for Seed per plants. Figure S7: Residual fitted model for Yield. Figure S8: Residual fitted model for Seed weight. Figure S9: Residual fitted model for Seed diameter. Figure S10: Residual fitted model for Seed thickness. [file PEI3-7-e70162-s002.docx]

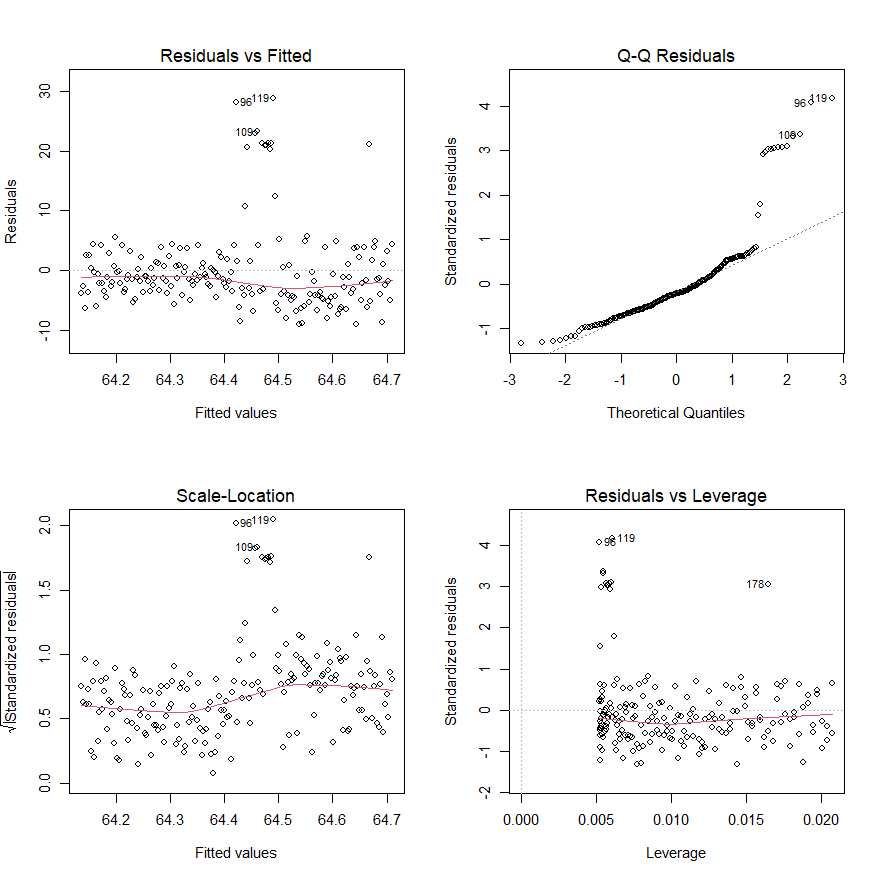


Figure_S1. Residual fitted model for Days to flowering


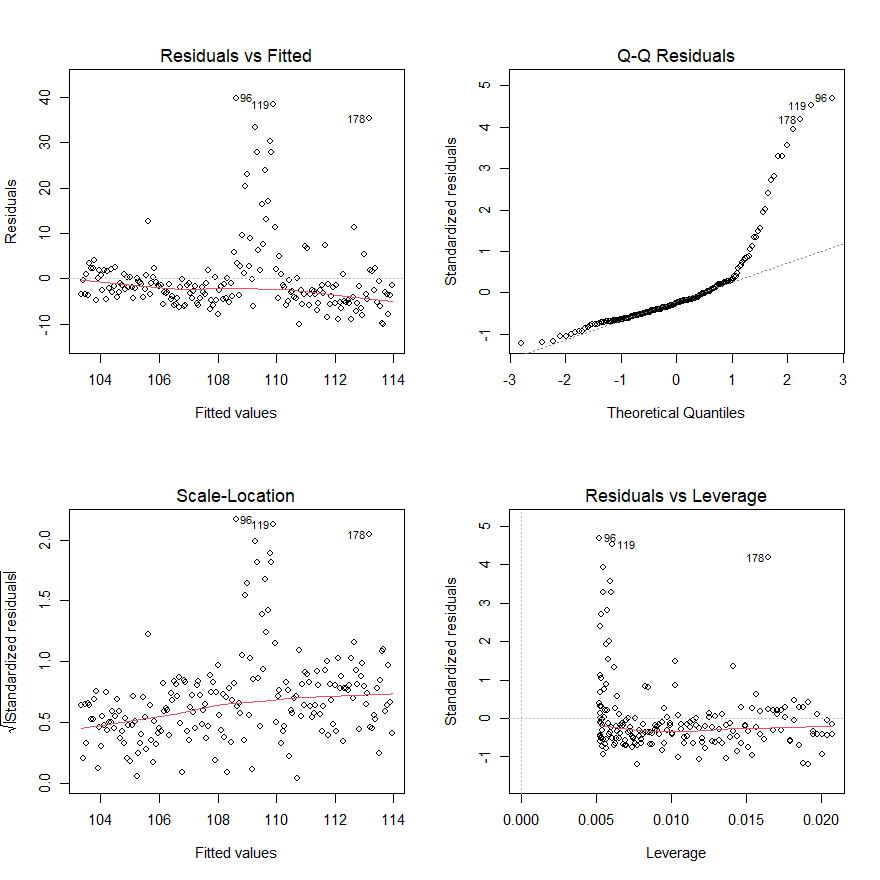


Figure_S2. Residual fitted model for Days to maturity


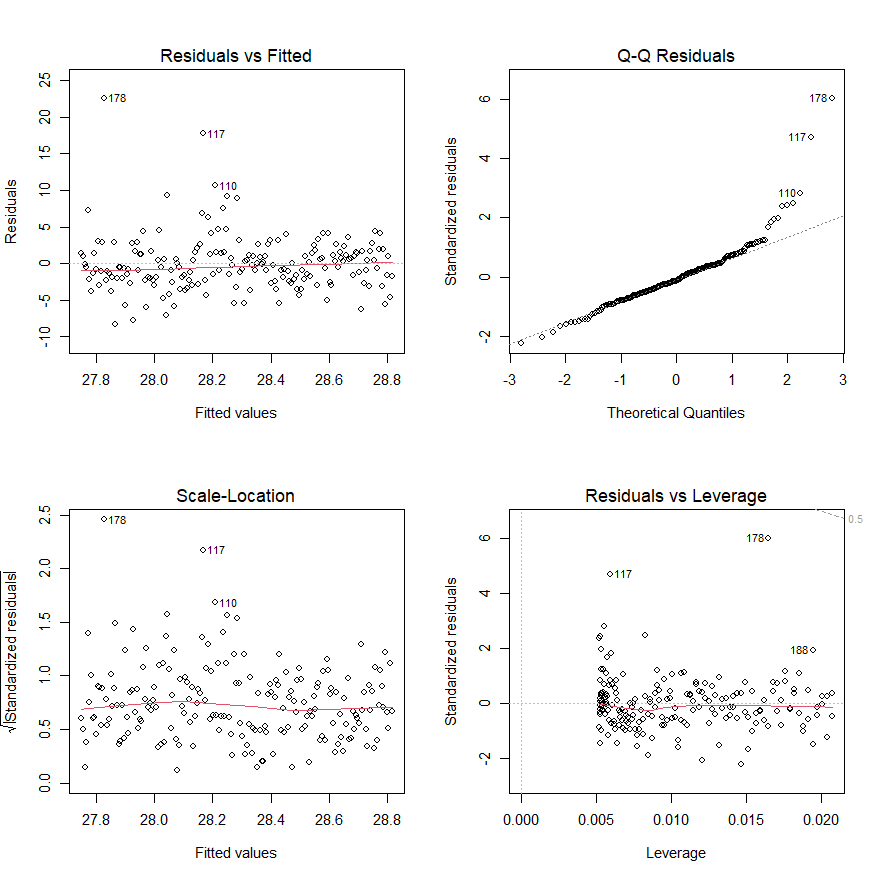


Figure_S3. Residual fitted model for Plant height


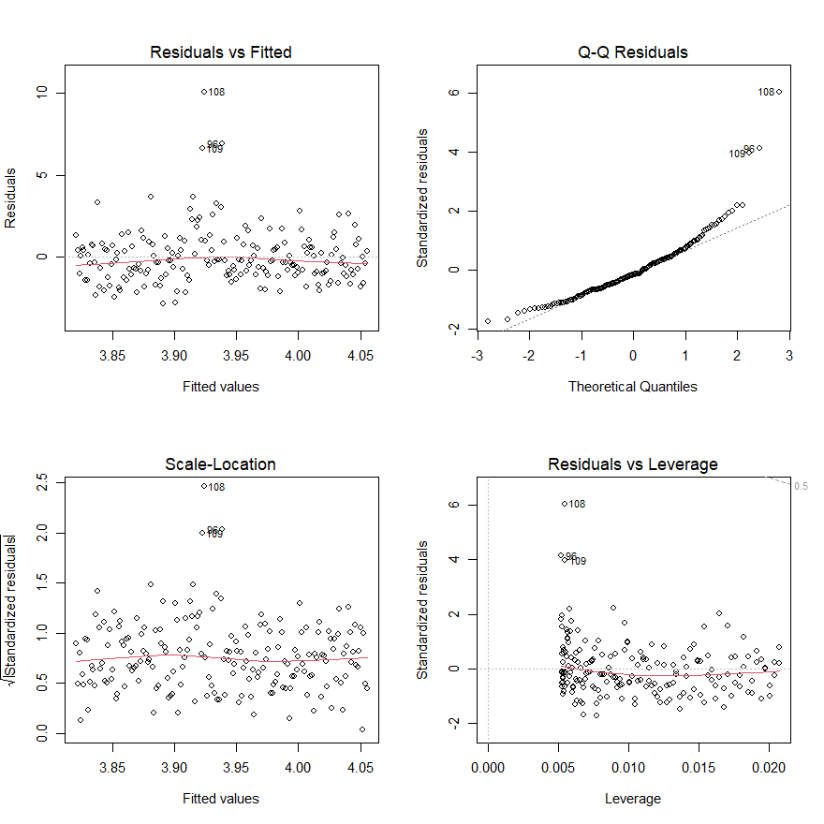


Figure_S4. Residual fitted model for Secondary branch per plant


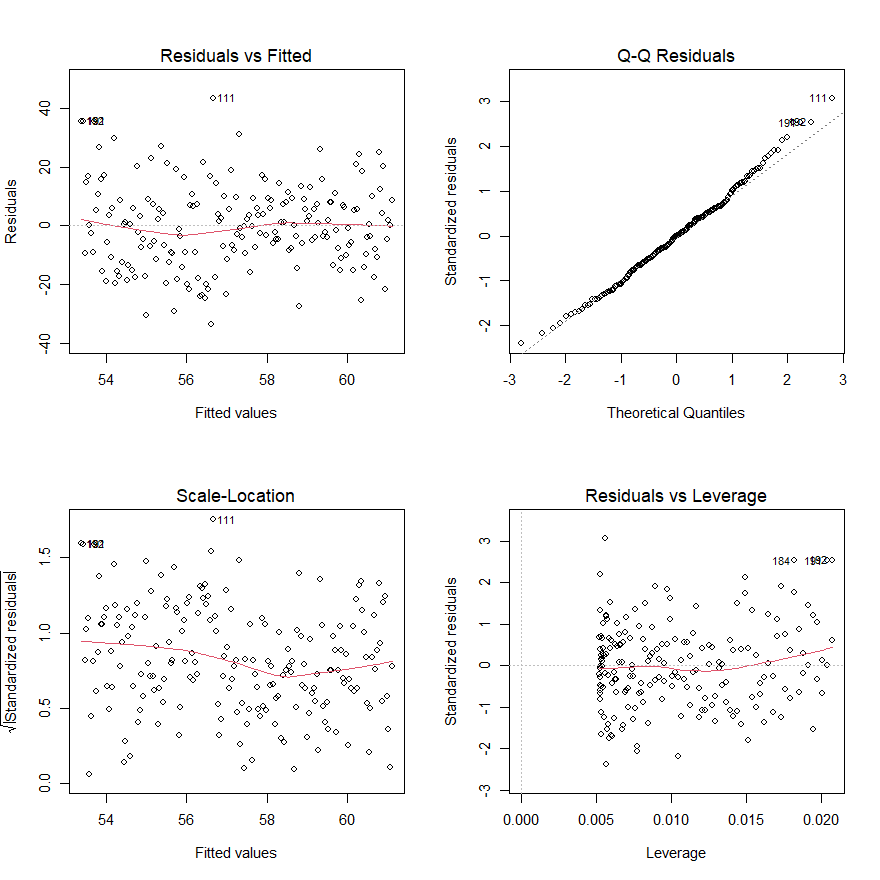


Figure_S5. Residual fitted model for Pod per plant


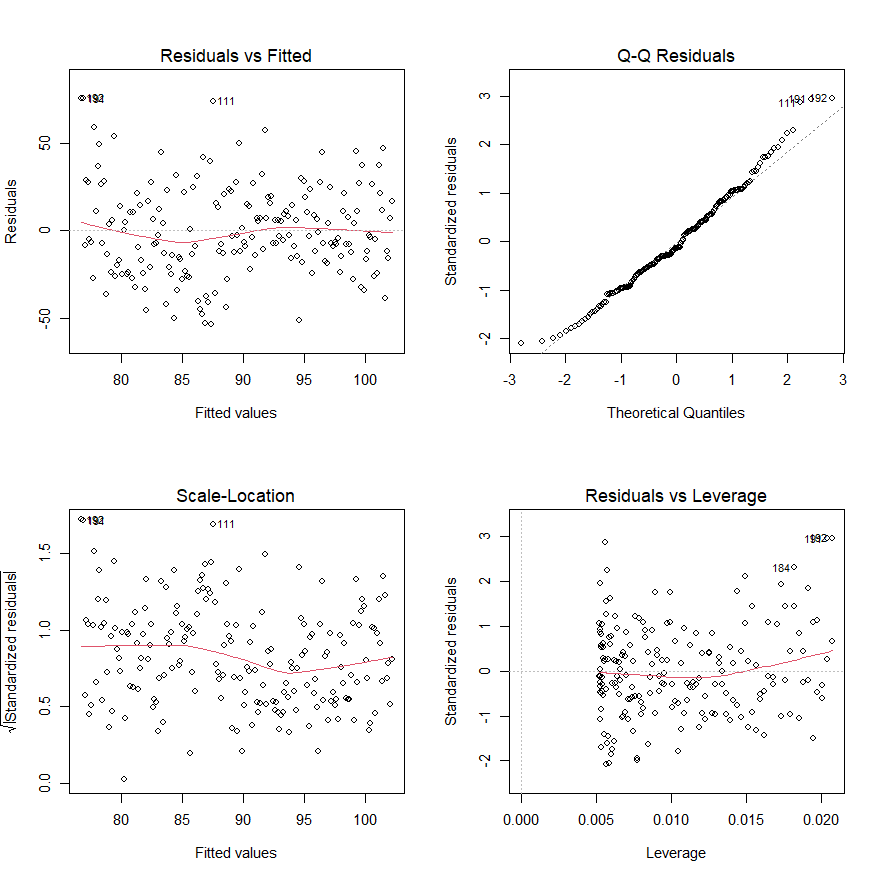


Figure_S6. Residual fitted model for Seed per plants


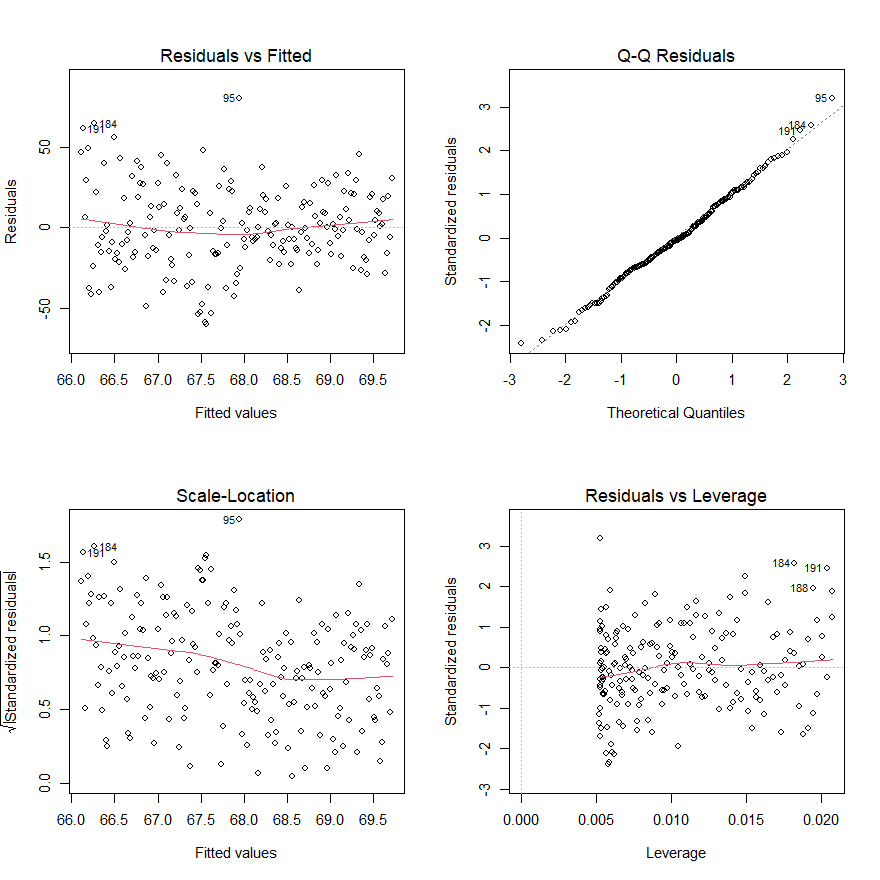


Figure_S7. Residual fitted model for Yield


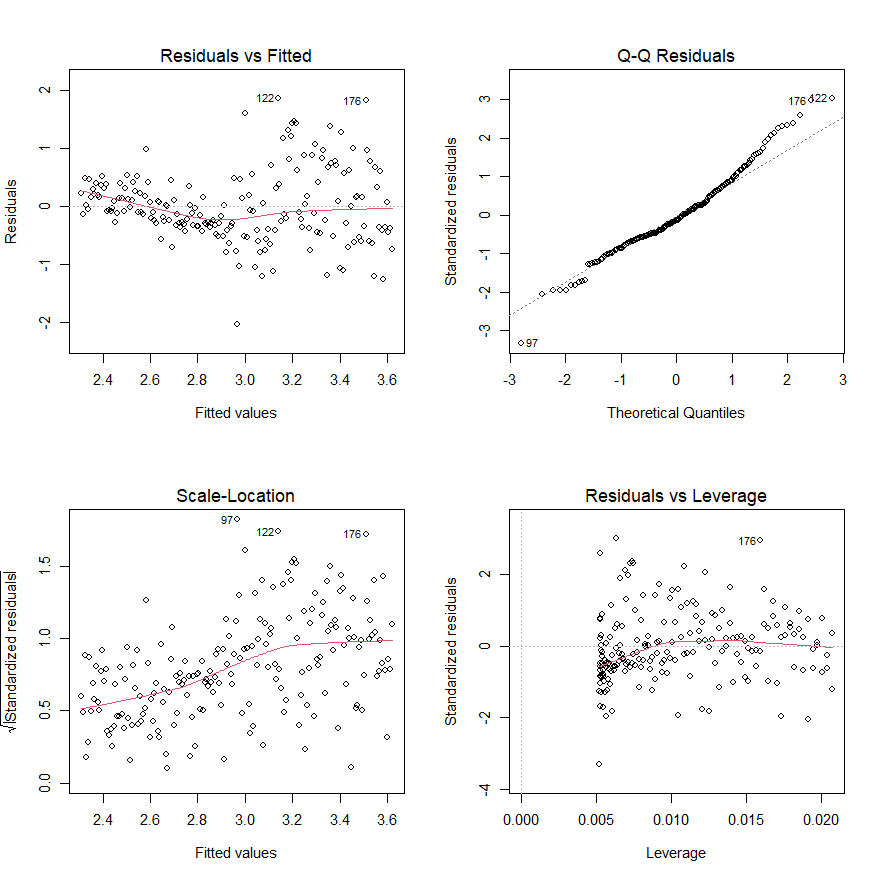


Figure_S8. Residual fitted model for Seed weight


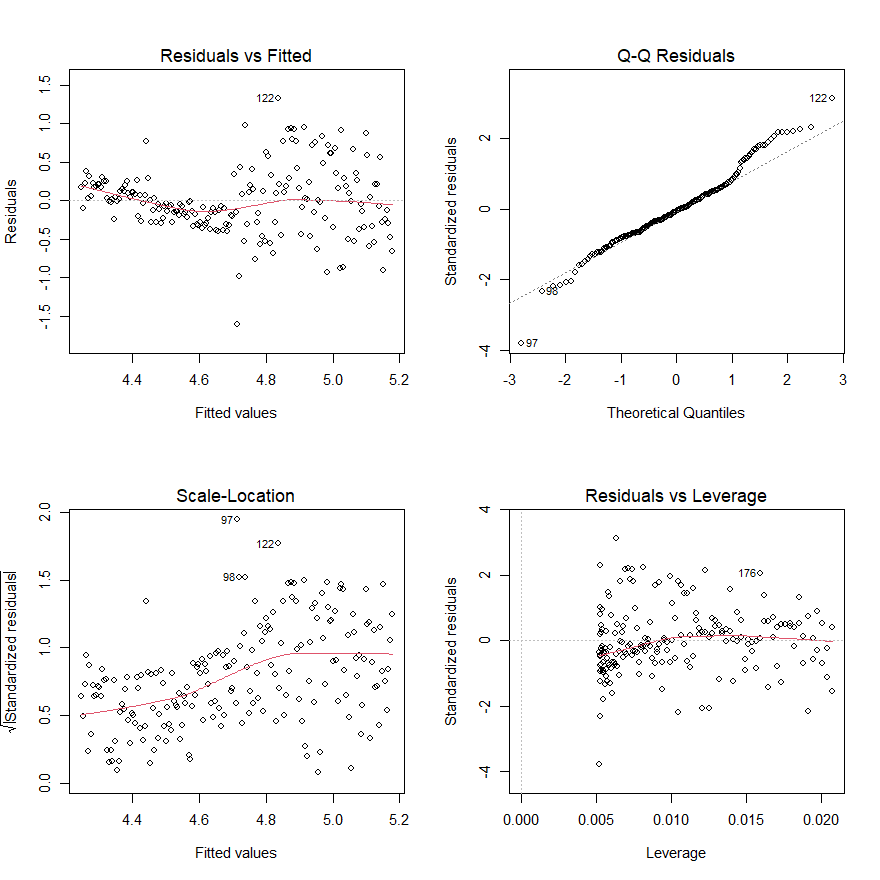


Figure_S9. Residual fitted model for Seed diameter


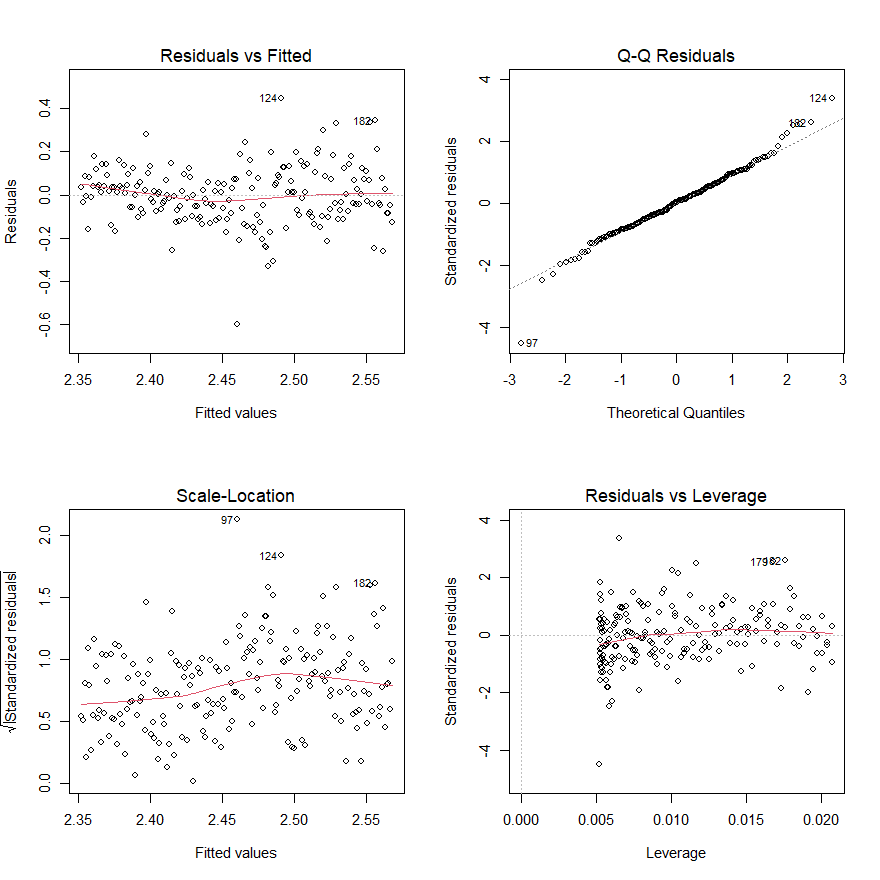


Figure_S10. Residual fitted model for Seed thickness
