# Supplementary material for: Association of Qualitative Characters With Agronomic Traits, and Their Breeding Importance in Lentil (Lens culinaris Medikus)
Source: Plant Environ Interact. 2026 May 13;7(3):e70162. doi: 10.1002/pei3.70162 (PMC13172294; doi:10.1002/pei3.70162)
Supplement: Supplementary file 2 — Table S1: List of planting material (germplasm) by their source of origin. Table S2: Contingency table of categorical variables. Table S3: The first five principal components for both experiments. [file PEI3-7-e70162-s003.docx]

∙

Table S1. List of planting material (germplasm) by their source of origin

| Population by Source | Germplasm | Sources |
| --- | --- | --- |
| ICARDA | 100, 122, 123, 135, 14, 140, 147, 171, 175, 185, 206, 207, 211161, 211169, 211171, 211174, 211182, 218, 219, 221, 230, 234, 239, 250, 254, 255, 269, 276, 277, 288, 297, 299, 3, 306, 315, 320, 324, 331, 336, 348, 350, 353, 36, 366, 374, 380, 386, 393, 394, 399, 407, 415, 416, 418, 429, 446, 447, 460, 72, 81, 86, 92 | Donated from International Center for Agricultural Research in Dry Areas |
| ACIAR | L-1, L-11, L-2, L-20, L-21, L-23, L-24, L-26, L-30, L-33, L-34, L-4, L-42, L-43, L-45, L-47, L-49, L-50, L-54, L-55, L-56, L-60, L-7, L-8, L-9 | Donated from Australian Center for International Agricultural Research |
| Amara | 16633, 16635, 17992, 17996, 207257, 207259, 207269, 207275, 207291, 207292, 207299, 207301, 207304, 207305, 207309, 228242, 229179, 229183, 235011, 235013, 235017, 236484, 238979, 241781, 243436, 243440, 243441, 244603, 244608, 244611, 244622, 24837, 24839, 26642 | Ethiopia Biodiversity Institute |
| Oromiya | 16527, 16528, 16529, 16530, 16531, 17731, 208757, 212851, 215488, 223323, 228243, 230019, 230520, 230521, 230522, 230833, 230835, 230836, 231244, 231251, 235585, 237024, 237026, 244803, 26985, 28744, 28745, 28748, 29797, 36147 | Ethiopia Biodiversity Institute |
| Tigray | 207263, 207265, 207901, 213258, 221720, 233663, 233664, 233665, 233668, 233669, 235383, 235385, 235389, 235390, 237504, 238274, 241785, 242602, 243446, 243447, 26066, 26068, 26070, 26071, 26072, 26073 | Ethiopia Biodiversity Institute |
| Varieties | Ada'a, Alem Tena, Alemaya, Assano, Beredu, Chekole, Debine, Denbi, Derash, Furi, Gudo, Jiru, Local, Teshale, chalew | Debre Zeit Agricultural Research Center |

Table S2. Contingency table of categorical variables

| Variable1 | Variable2 | Level_var1 | Level_var2 | Count |
| --- | --- | --- | --- | --- |
| EPV | SSP | 1 | 0 | 20 |
| EPV | SSP | 2 | 0 | 38 |
| EPV | SSP | 3 | 0 | 23 |
| EPV | SSP | 1 | 1 | 30 |
| EPV | SSP | 2 | 1 | 51 |
| EPV | SSP | 3 | 1 | 30 |
| EPV | GH | 1 | 0 | 24 |
| EPV | GH | 2 | 0 | 54 |
| EPV | GH | 3 | 0 | 35 |
| EPV | GH | 1 | 1 | 26 |
| EPV | GH | 2 | 1 | 35 |
| EPV | GH | 3 | 1 | 18 |
| EPV | LC | 1 | 0 | 31 |
| EPV | LC | 2 | 0 | 56 |
| EPV | LC | 3 | 0 | 35 |
| EPV | LC | 1 | 1 | 19 |
| EPV | LC | 2 | 1 | 33 |
| EPV | LC | 3 | 1 | 18 |
| EPV | LP | 1 | 0 | 14 |
| EPV | LP | 2 | 0 | 20 |
| EPV | LP | 3 | 0 | 15 |
| EPV | LP | 1 | 3 | 20 |
| EPV | LP | 2 | 3 | 44 |
| EPV | LP | 3 | 3 | 23 |
| EPV | LP | 1 | 5 | 16 |
| EPV | LP | 2 | 5 | 25 |
| EPV | LP | 3 | 5 | 15 |
| EPV | LS | 1 | 3 | 31 |
| EPV | LS | 2 | 3 | 33 |
| EPV | LS | 3 | 3 | 14 |
| EPV | LS | 1 | 5 | 19 |
| EPV | LS | 2 | 5 | 56 |
| EPV | LS | 3 | 5 | 39 |
| EPV | FGC | 1 | 0 | 15 |
| EPV | FGC | 2 | 0 | 29 |
| EPV | FGC | 3 | 0 | 21 |
| EPV | FGC | 1 | 1 | 35 |
| EPV | FGC | 2 | 1 | 60 |
| EPV | FGC | 3 | 1 | 32 |
| EPV | LoD | 1 | 0 | 33 |
| EPV | LoD | 2 | 0 | 40 |
| EPV | LoD | 3 | 0 | 15 |
| EPV | LoD | 1 | 1 | 17 |
| EPV | LoD | 2 | 1 | 49 |
| EPV | LoD | 3 | 1 | 38 |
| EPV | PP | 1 | 0 | 37 |
| EPV | PP | 2 | 0 | 59 |
| EPV | PP | 3 | 0 | 31 |
| EPV | PP | 1 | 1 | 13 |
| EPV | PP | 2 | 1 | 30 |
| EPV | PP | 3 | 1 | 22 |
| EPV | SCC | 1 | 2 | 16 |
| EPV | SCC | 2 | 2 | 25 |
| EPV | SCC | 3 | 2 | 21 |
| EPV | SCC | 1 | 3 | 25 |
| EPV | SCC | 2 | 3 | 50 |
| EPV | SCC | 3 | 3 | 27 |
| EPV | SCC | 1 | 5 | 9 |
| EPV | SCC | 2 | 5 | 14 |
| EPV | SCC | 3 | 5 | 5 |
| EPV | PST | 1 | 0 | 13 |
| EPV | PST | 2 | 0 | 29 |
| EPV | PST | 3 | 0 | 22 |
| EPV | PST | 1 | 2 | 7 |
| EPV | PST | 2 | 2 | 10 |
| EPV | PST | 3 | 2 | 6 |
| EPV | PST | 1 | 4 | 30 |
| EPV | PST | 2 | 4 | 50 |
| EPV | PST | 3 | 4 | 27 |
| EPV | CC | 1 | 0 | 6 |
| EPV | CC | 2 | 0 | 12 |
| EPV | CC | 3 | 0 | 16 |
| EPV | CC | 1 | 1 | 47 |
| EPV | CC | 2 | 1 | 77 |
| EPV | CC | 3 | 1 | 37 |
| SSP | GH | 0 | 0 | 48 |
| SSP | GH | 1 | 0 | 65 |
| SSP | GH | 0 | 1 | 33 |
| SSP | GH | 1 | 1 | 46 |
| SSP | LC | 0 | 0 | 51 |
| SSP | LC | 1 | 0 | 71 |
| SSP | LC | 0 | 1 | 30 |
| SSP | LC | 1 | 1 | 40 |
| SSP | LP | 0 | 0 | 26 |
| SSP | LP | 1 | 0 | 23 |
| SSP | LP | 0 | 3 | 42 |
| SSP | LP | 1 | 3 | 45 |
| SSP | LP | 0 | 5 | 13 |
| SSP | LP | 1 | 5 | 43 |
| SSP | LS | 0 | 3 | 33 |
| SSP | LS | 1 | 3 | 45 |
| SSP | LS | 0 | 5 | 48 |
| SSP | LS | 1 | 5 | 66 |
| SSP | FGC | 0 | 0 | 25 |
| SSP | FGC | 1 | 0 | 40 |
| SSP | FGC | 0 | 1 | 56 |
| SSP | FGC | 1 | 1 | 71 |
| SSP | LoD | 0 | 0 | 29 |
| SSP | LoD | 1 | 0 | 59 |
| SSP | LoD | 0 | 1 | 52 |
| SSP | LoD | 1 | 1 | 52 |
| SSP | PP | 0 | 0 | 60 |
| SSP | PP | 1 | 0 | 67 |
| SSP | PP | 0 | 1 | 21 |
| SSP | PP | 1 | 1 | 44 |
| SSP | SCC | 0 | 2 | 21 |
| SSP | SCC | 1 | 2 | 41 |
| SSP | SCC | 0 | 3 | 47 |
| SSP | SCC | 1 | 3 | 55 |
| SSP | SCC | 0 | 5 | 13 |
| SSP | SCC | 1 | 5 | 15 |
| SSP | PST | 0 | 0 | 24 |
| SSP | PST | 1 | 0 | 40 |
| SSP | PST | 0 | 2 | 8 |
| SSP | PST | 1 | 2 | 13 |
| SSP | PST | 0 | 4 | 49 |
| SSP | PST | 1 | 4 | 58 |
| SSP | CC | 0 | 0 | 7 |
| SSP | CC | 1 | 0 | 24 |
| SSP | CC | 0 | 1 | 74 |
| SSP | CC | 1 | 1 | 87 |
| GH | LC | 0 | 0 | 65 |
| GH | LC | 1 | 0 | 57 |
| GH | LC | 0 | 1 | 48 |
| GH | LC | 1 | 1 | 22 |
| GH | LP | 0 | 0 | 32 |
| GH | LP | 1 | 0 | 17 |
| GH | LP | 0 | 3 | 48 |
| GH | LP | 1 | 3 | 39 |
| GH | LP | 0 | 5 | 33 |
| GH | LP | 1 | 5 | 23 |
| GH | LS | 0 | 3 | 43 |
| GH | LS | 1 | 3 | 35 |
| GH | LS | 0 | 5 | 70 |
| GH | LS | 1 | 5 | 44 |
| GH | FGC | 0 | 0 | 50 |
| GH | FGC | 1 | 0 | 15 |
| GH | FGC | 0 | 1 | 63 |
| GH | FGC | 1 | 1 | 64 |
| GH | LoD | 0 | 0 | 55 |
| GH | LoD | 1 | 0 | 33 |
| GH | LoD | 0 | 1 | 58 |
| GH | LoD | 1 | 1 | 46 |
| GH | PP | 0 | 0 | 66 |
| GH | PP | 1 | 0 | 61 |
| GH | PP | 0 | 1 | 47 |
| GH | PP | 1 | 1 | 18 |
| GH | SCC | 0 | 2 | 43 |
| GH | SCC | 1 | 2 | 19 |
| GH | SCC | 0 | 3 | 48 |
| GH | SCC | 1 | 3 | 54 |
| GH | SCC | 0 | 5 | 22 |
| GH | SCC | 1 | 5 | 6 |
| GH | PST | 0 | 0 | 48 |
| GH | PST | 1 | 0 | 16 |
| GH | PST | 0 | 2 | 15 |
| GH | PST | 1 | 2 | 6 |
| GH | PST | 0 | 4 | 50 |
| GH | PST | 1 | 4 | 57 |
| GH | CC | 0 | 0 | 23 |
| GH | CC | 1 | 0 | 8 |
| GH | CC | 0 | 1 | 90 |
| GH | CC | 1 | 1 | 71 |
| LC | LP | 0 | 0 | 31 |
| LC | LP | 1 | 0 | 18 |
| LC | LP | 0 | 3 | 52 |
| LC | LP | 1 | 3 | 35 |
| LC | LP | 0 | 5 | 39 |
| LC | LP | 1 | 5 | 17 |
| LC | LS | 0 | 3 | 53 |
| LC | LS | 1 | 3 | 25 |
| LC | LS | 0 | 5 | 69 |
| LC | LS | 1 | 5 | 45 |
| LC | FGC | 0 | 0 | 29 |
| LC | FGC | 1 | 0 | 36 |
| LC | FGC | 0 | 1 | 93 |
| LC | FGC | 1 | 1 | 34 |
| LC | LoD | 0 | 0 | 51 |
| LC | LoD | 1 | 0 | 37 |
| LC | LoD | 0 | 1 | 71 |
| LC | LoD | 1 | 1 | 33 |
| LC | PP | 0 | 0 | 86 |
| LC | PP | 1 | 0 | 41 |
| LC | PP | 0 | 1 | 36 |
| LC | PP | 1 | 1 | 29 |
| LC | SCC | 0 | 2 | 37 |
| LC | SCC | 1 | 2 | 25 |
| LC | SCC | 0 | 3 | 76 |
| LC | SCC | 1 | 3 | 26 |
| LC | SCC | 0 | 5 | 9 |
| LC | SCC | 1 | 5 | 19 |
| LC | PST | 0 | 0 | 30 |
| LC | PST | 1 | 0 | 34 |
| LC | PST | 0 | 2 | 11 |
| LC | PST | 1 | 2 | 10 |
| LC | PST | 0 | 4 | 81 |
| LC | PST | 1 | 4 | 26 |
| LC | CC | 0 | 0 | 17 |
| LC | CC | 1 | 0 | 14 |
| LC | CC | 0 | 1 | 105 |
| LC | CC | 1 | 1 | 56 |
| LP | LS | 0 | 3 | 31 |
| LP | LS | 3 | 3 | 35 |
| LP | LS | 5 | 3 | 12 |
| LP | LS | 0 | 5 | 18 |
| LP | LS | 3 | 5 | 52 |
| LP | LS | 5 | 5 | 44 |
| LP | FGC | 0 | 0 | 7 |
| LP | FGC | 3 | 0 | 26 |
| LP | FGC | 5 | 0 | 32 |
| LP | FGC | 0 | 1 | 42 |
| LP | FGC | 3 | 1 | 61 |
| LP | FGC | 5 | 1 | 24 |
| LP | LoD | 0 | 0 | 23 |
| LP | LoD | 3 | 0 | 34 |
| LP | LoD | 5 | 0 | 31 |
| LP | LoD | 0 | 1 | 26 |
| LP | LoD | 3 | 1 | 53 |
| LP | LoD | 5 | 1 | 25 |
| LP | PP | 0 | 0 | 34 |
| LP | PP | 3 | 0 | 60 |
| LP | PP | 5 | 0 | 33 |
| LP | PP | 0 | 1 | 15 |
| LP | PP | 3 | 1 | 27 |
| LP | PP | 5 | 1 | 23 |
| LP | SCC | 0 | 2 | 5 |
| LP | SCC | 3 | 2 | 21 |
| LP | SCC | 5 | 2 | 36 |
| LP | SCC | 0 | 3 | 39 |
| LP | SCC | 3 | 3 | 52 |
| LP | SCC | 5 | 3 | 11 |
| LP | SCC | 0 | 5 | 5 |
| LP | SCC | 3 | 5 | 14 |
| LP | SCC | 5 | 5 | 9 |
| LP | PST | 0 | 0 | 5 |
| LP | PST | 3 | 0 | 26 |
| LP | PST | 5 | 0 | 33 |
| LP | PST | 0 | 2 | 5 |
| LP | PST | 3 | 2 | 8 |
| LP | PST | 5 | 2 | 8 |
| LP | PST | 0 | 4 | 39 |
| LP | PST | 3 | 4 | 53 |
| LP | PST | 5 | 4 | 15 |
| LP | CC | 0 | 0 | 6 |
| LP | CC | 3 | 0 | 13 |
| LP | CC | 5 | 0 | 17 |
| LP | CC | 0 | 1 | 48 |
| LP | CC | 3 | 1 | 74 |
| LP | CC | 5 | 1 | 39 |
| LS | FGC | 3 | 0 | 14 |
| LS | FGC | 5 | 0 | 51 |
| LS | FGC | 3 | 1 | 64 |
| LS | FGC | 5 | 1 | 63 |
| LS | LoD | 3 | 0 | 34 |
| LS | LoD | 5 | 0 | 54 |
| LS | LoD | 3 | 1 | 44 |
| LS | LoD | 5 | 1 | 60 |
| LS | PP | 3 | 0 | 57 |
| LS | PP | 5 | 0 | 70 |
| LS | PP | 3 | 1 | 21 |
| LS | PP | 5 | 1 | 44 |
| LS | SCC | 3 | 2 | 13 |
| LS | SCC | 5 | 2 | 49 |
| LS | SCC | 3 | 3 | 55 |
| LS | SCC | 5 | 3 | 47 |
| LS | SCC | 3 | 5 | 10 |
| LS | SCC | 5 | 5 | 18 |
| LS | PST | 3 | 0 | 11 |
| LS | PST | 5 | 0 | 53 |
| LS | PST | 3 | 2 | 9 |
| LS | PST | 5 | 2 | 12 |
| LS | PST | 3 | 4 | 58 |
| LS | PST | 5 | 4 | 49 |
| LS | CC | 3 | 0 | 6 |
| LS | CC | 5 | 0 | 27 |
| LS | CC | 3 | 1 | 74 |
| LS | CC | 5 | 1 | 87 |
| FGC | LoD | 0 | 0 | 37 |
| FGC | LoD | 1 | 0 | 51 |
| FGC | LoD | 0 | 1 | 28 |
| FGC | LoD | 1 | 1 | 76 |
| FGC | PP | 0 | 0 | 31 |
| FGC | PP | 1 | 0 | 96 |
| FGC | PP | 0 | 1 | 34 |
| FGC | PP | 1 | 1 | 31 |
| FGC | SCC | 0 | 2 | 42 |
| FGC | SCC | 1 | 2 | 20 |
| FGC | SCC | 0 | 3 | 7 |
| FGC | SCC | 1 | 3 | 95 |
| FGC | SCC | 0 | 5 | 16 |
| FGC | SCC | 1 | 5 | 12 |
| FGC | PST | 0 | 0 | 54 |
| FGC | PST | 1 | 0 | 10 |
| FGC | PST | 0 | 2 | 5 |
| FGC | PST | 1 | 2 | 16 |
| FGC | PST | 0 | 4 | 6 |
| FGC | PST | 1 | 4 | 101 |
| FGC | CC | 0 | 0 | 22 |
| FGC | CC | 1 | 0 | 9 |
| FGC | CC | 0 | 1 | 43 |
| FGC | CC | 1 | 1 | 118 |
| LoD | PP | 0 | 0 | 55 |
| LoD | PP | 1 | 0 | 72 |
| LoD | PP | 0 | 1 | 33 |
| LoD | PP | 1 | 1 | 32 |
| LoD | SCC | 0 | 2 | 33 |
| LoD | SCC | 1 | 2 | 29 |
| LoD | SCC | 0 | 3 | 35 |
| LoD | SCC | 1 | 3 | 67 |
| LoD | SCC | 0 | 5 | 20 |
| LoD | SCC | 1 | 5 | 8 |
| LoD | PST | 0 | 0 | 37 |
| LoD | PST | 1 | 0 | 27 |
| LoD | PST | 0 | 2 | 12 |
| LoD | PST | 1 | 2 | 9 |
| LoD | PST | 0 | 4 | 39 |
| LoD | PST | 1 | 4 | 68 |
| LoD | CC | 0 | 0 | 16 |
| LoD | CC | 1 | 0 | 15 |
| LoD | CC | 0 | 1 | 72 |
| LoD | CC | 1 | 1 | 89 |
| PP | SCC | 0 | 2 | 30 |
| PP | SCC | 1 | 2 | 32 |
| PP | SCC | 0 | 3 | 83 |
| PP | SCC | 1 | 3 | 19 |
| PP | SCC | 0 | 5 | 14 |
| PP | SCC | 1 | 5 | 14 |
| PP | PST | 0 | 0 | 27 |
| PP | PST | 1 | 0 | 37 |
| PP | PST | 0 | 2 | 13 |
| PP | PST | 1 | 2 | 8 |
| PP | PST | 0 | 4 | 87 |
| PP | PST | 1 | 4 | 20 |
| PP | CC | 0 | 0 | 12 |
| PP | CC | 1 | 0 | 19 |
| PP | CC | 0 | 1 | 115 |
| PP | CC | 1 | 1 | 46 |
| SCC | PST | 2 | 0 | 44 |
| SCC | PST | 3 | 0 | 6 |
| SCC | PST | 5 | 0 | 18 |
| SCC | PST | 2 | 2 | 10 |
| SCC | PST | 3 | 2 | 6 |
| SCC | PST | 5 | 2 | 10 |
| SCC | PST | 2 | 4 | 8 |
| SCC | PST | 3 | 4 | 99 |
| SCC | PST | 5 | 4 | 6 |
| SCC | CC | 2 | 0 | 25 |
| SCC | CC | 3 | 0 | 6 |
| SCC | CC | 5 | 0 | 5 |
| SCC | CC | 2 | 1 | 37 |
| SCC | CC | 3 | 1 | 101 |
| SCC | CC | 5 | 1 | 23 |
| PST | CC | 0 | 0 | 24 |
| PST | CC | 2 | 0 | 6 |
| PST | CC | 4 | 0 | 6 |
| PST | CC | 0 | 1 | 40 |
| PST | CC | 2 | 1 | 15 |
| PST | CC | 4 | 1 | 106 |

EPV - early plant vigor, LP – Leaf pubescence, LS – Leaf size, SSP – Seedling stem pigmentation, GH – Growth habit, LC – leaf color, FGC – Flower ground color, PP – pod pigmentation, CC – cotyledon color, SCC – Seed coat color, PST – Pattern of seed testa,

Table S3. The first five principal components for both experiments

| Field exp. | | | | | | Pot exp. | | | | |
| --- | --- | --- | --- | --- | --- | --- | --- | --- | --- | --- |
| Traits | PC1 | PC2 | PC3 | PC4 | PC5 | PC1 | PC2 | PC3 | PC4 | PC5 |
| EPV | 0.11 | -0.06 | -0.06 | 0.48 | 0.15 | 0.05 | -0.19 | 0.08 | 0.42 | -0.35 |
| LC | 0.09 | -0.03 | 0.05 | -0.36 | 0.26 | 0.06 | -0.02 | -0.06 | -0.41 | -0.41 |
| LP | 0.2 | 0.11 | 0.13 | 0.02 | -0.33 | 0.19 | 0.01 | 0.24 | -0.07 | 0.29 |
| LS | 0.19 | 0.06 | 0.12 | 0.17 | -0.02 | 0.14 | -0.1 | 0.28 | 0.04 | -0.13 |
| SSP | 0.08 | 0.03 | -0.02 | -0.07 | -0.49 | 0.07 | -0.02 | 0.04 | -0.12 | 0.2 |
| GH | -0.15 | 0.14 | 0.05 | 0.08 | -0.32 | -0.08 | 0.22 | 0.08 | 0.1 | 0.27 |
| FGC | -0.34 | 0.09 | -0.08 | 0.18 | -0.02 | -0.28 | 0.19 | 0.03 | 0.19 | 0.04 |
| LoD | -0.07 | 0.03 | -0.04 | 0.52 | 0.27 | -0.09 | -0.05 | 0.08 | 0.45 | -0.17 |
| PP | 0.2 | -0.07 | -0.1 | -0.05 | -0.06 | 0.1 | -0.24 | 0.06 | -0.22 | -0.14 |
| CC | -0.27 | -0.05 | -0.14 | -0.16 | 0.15 | -0.24 | 0.11 | -0.23 | -0.07 | 0.05 |
| SCC | -0.09 | -0.01 | 0.02 | -0.39 | 0.38 | -0.08 | 0.06 | -0.11 | -0.29 | -0.33 |
| PST | -0.36 | 0.03 | -0.14 | 0.18 | -0.02 | -0.3 | 0.15 | -0.06 | 0.26 | 0.09 |
| DF | -0.04 | 0.24 | 0.43 | -0.01 | 0.15 | 0.15 | 0.33 | 0.36 | -0.05 | 0.02 |
| DM | 0.09 | 0.18 | 0.46 | -0.01 | 0.07 | 0.22 | 0.28 | 0.31 | -0.07 | 0.14 |
| PH | -0.07 | -0.13 | 0.37 | 0.21 | 0.22 | 0.03 | -0.02 | 0.35 | 0.26 | -0.36 |
| SBPP | -0.14 | 0.02 | 0.4 | -0.04 | -0.21 | -0.07 | -0.07 | 0.38 | -0.18 | 0.08 |
| PPP | -0.24 | -0.3 | 0.17 | -0.1 | -0.15 | -0.35 | -0.09 | 0.15 | -0.13 | -0.02 |
| SPP | -0.3 | -0.28 | 0.14 | -0.09 | -0.13 | -0.37 | -0.04 | 0.15 | -0.1 | 0.03 |
| BM | -0.05 | -0.36 | 0.3 | 0.11 | 0.01 | -0.17 | -0.25 | 0.4 | -0.03 | 0.13 |
| SW | 0.36 | -0.24 | 0.02 | 0.05 | -0.01 | 0.29 | -0.32 | -0.05 | 0.08 | 0.12 |
| YLD | -0.06 | -0.48 | 0.12 | 0.05 | -0.04 | -0.25 | -0.37 | 0.13 | -0.07 | 0.14 |
| HI | -0.04 | -0.42 | -0.22 | 0.02 | -0.07 | -0.25 | -0.37 | -0.02 | -0.08 | 0.12 |
| SD | 0.38 | -0.18 | 0.05 | 0.05 | -0.07 | 0.28 | -0.32 | -0.02 | 0.06 | 0.09 |
| ST | 0.22 | -0.19 | -0.01 | -0.06 | 0.2 | 0.15 | -0.18 | -0.24 | 0.15 | 0.31 |
| SD | 2.25 | 1.9 | 1.82 | 1.27 | 1.18 | 2.51 | 1.85 | 1.51 | 1.28 | 1.22 |
| Proportion | 0.21 | 0.15 | 0.14 | 0.07 | 0.06 | 0.26 | 0.14 | 0.1 | 0.07 | 0.06 |
| Cumulative | 0.21 | 0.36 | 0.5 | 0.57 | 0.62 | 0.26 | 0.41 | 0.5 | 0.57 | 0.63 |

EPV - early plant vigor, LP – Leaf pubescence, LS – Leaf size, SSP – Seedling stem pigmentation, GH – Growth habit, LC – leaf color, FGC – Flower ground color, PP – pod pigmentation, CC – cotyledon color, SCC – Seed coat color, PST – Pattern of seed testa, DF – days to flowering, DM – days to maturity, SBPP – number of secondary branches per plant, PPP – number of pods per plant, SPP – number of seeds per plant, BM – biomass yield in gram per plot for field and per plant for pot experiment, YLD – yield in gram per plot for field and per plant for pot experiment, HI – Harvest index, SW – hundred seed weight in gram, SD – seed diameter in mm, ST – seed thickness in mm
